# Supplementary material for: Cerebrospinal fluid reference proteins increase accuracy and interpretability of biomarkers for brain diseases
Source: Nat Commun. 2024 May 1;15:3676. doi: 10.1038/s41467-024-47971-5 (PMC11063138; doi:10.1038/s41467-024-47971-5)
Supplement: Supplementary file 1 — Supplementary Information [file 41467_2024_47971_MOESM1_ESM.pdf]

Supplementary Information

Cerebrospinal fluid reference proteins increase accuracy and interpretability of biomarkers for brain diseases

Supplementary Tables

Supplementary Table 1: Predicting the mean CSF protein level in a multiple linear regression model.

Supplementary Table 2: Cluster 11 consisted of proteins that are more highly expressed in the brain compared to the other highly expressed CSF proteins.

Supplementary Table 3: Performance details for all models with and without references.

Supplementary Table 4: Effect sizes of association with CSF P-tau181 for sTREM2, sAXL, sTyro3 and YKL-40 without and with reference proteins.

Supplementary Table 5: Effect sizes of association with CSF P-tau181 for α-synuclein without and with reference proteins.

Supplementary Table 6: Effect sizes of *APOE* ε4 allele association with ApoE4 CSF protein expression without and with reference proteins.

Supplementary Table 7: The selected pQTL-analysis proteins and their association with the mean CSF level.

Supplementary Table 8: Effect of genotype (SNP) in associations with CSF proteins without and with reference proteins.

Supplementary Table 9: OLINK Panel statistics.

Supplementary Table 10: Association with the mean CSF level for the proteins that were analyzed.

Supplementary Figures

Supplementary Figure 1: Absolute association (β-value) with mean CSF level for all 2944 proteins.

Supplementary Figure 2: Visualization of individual CSF levels stratified by age.

Supplementary Figure 3: Visualization of individual CSF levels stratified by clinical status.

Supplementary Figure 4: Visualization of individual CSF levels only including proteins with missing frequency < 75%.

Supplementary Figure 5: t-SNE OLINK panel coloring.

Supplementary Figure 6: **Cluster 11 cell type expression.**

Supplementary Figure 7: **Cluster 11 cellular component enrichment analysis.**

Supplementary Figure 8: Correlation matrix of general candidates.

Supplementary Figure 9: sTREM2 levels by AT(N) grouping for NC, SCD and MCI.

Supplementary Figure 10: sAXL levels by AT(N) grouping for NC, SCD and MCI.

Supplementary Figure 11: sTyro3 levels by AT(N) grouping for NC, SCD and MCI.

Supplementary Figure 12: Correlation matrices for ten NeuroToolKit proteins in BF1 and BF2 with and without adjusting for a reference protein.

Supplementary Figure 13: Effects of the most common CSF *trans*-pQTL variant rs71635338 in associations with CSF proteins without and with reference protein NTRK3.

Supplementary Figure 14: Mean silhouette score for K-means clustering of t-SNE space.

Supplementary Figure 15: Semi-supervised K-means clustering of t-SNE space for different Ks and random initialization seeds.

Supplementary Figure 16: Location of the examined reference protein candidates in t-SNE map.

Supplementary Figure 17: Missing frequency t-SNE map.

Supplementary Figure 18: t-SNE on reduced data colored by panel.

Supplementary Figure 19: t-SNE on reduced data highlighting cluster 11.

Supplementary Figure 20: t-SNE on reduced data highlighting reference protein candidates.

Supplementary Figure 21: **Pipeline for pre-processing steps of all models.**

Supplementary Methods

OLINK Data Handling 27

Quality Control **..27**

Limit of Detection **..27**

LOD Sensitivity Analysis 27

K-means Robustness Analysis 28

Statistical Models 28

Linear Regression **..28**

Logistic Regression ……………………………………………………………………..…………29

Supplementary Results

Changes in results when adjusting for a reference protein ……………………………………..………30

NeuroToolKit correlations ………………………………………………………………………………..30

*APOE*4 genotype vs protein expression ……………………………………………………………..30

CSF pQTL analysis ………………………………………………………………………………………31

Adjusting for a Reference Protein in P-tau181 Applications ………………………………………………32

sTREM2, sAXL, sTyro3 and YKL-40 association with P-tau181 in NC, SCD and MCI ………………..…32

α-synuclein in AD Dementia ……………………………………………………………………………...33

Supplementary Note 1: Reference Protein Candidate Profiles

Neurotrophic receptor tyrosine kinase 3 (NTRK3) ……………………………………………..……..34

Neurotrophic receptor tyrosine kinase 2 (NTRK2) ………………………………………………………36

***Bleomycin hydrolase (BLMH)*…………………………………………………………………………38**

Cerebellin 4 precursor (CBLN4) ………………………………………………………………………40

Protein tyrosine phosphatase receptor type N2 (PTPRN2) …………………..………………………..42

Protein tyrosine phosphatase receptor type S (PTPRS) ………………………………………………44

Amyloid-β40 (Aβ40) ………………………………………………………………………………..46

Supplementary References …………………………..……………………………………48

Supplementary Table 1: Predicting the mean CSF protein level in a multiple linear regression model. Testing the association of independent variables age, sex, education years, intracranial volume, total gray matter volume and ventricular volume with the mean CSF protein level in a multiple linear regression model, with either all participants or cognitively unimpaired Aβ-negative participants only. Significant variables (two-sided P-value, not adjusted for multiple comparisons) are denoted with *.

	**Age**	**Sex**	**Education**	**Intracranial volume**	**Total gray matter volume**	**Ventricular volume**
All Participants (n=631)<br>R-squared = 0.256						
β-coef	0.544	-0.159	-0.018	0.037	0.018	-0.321
P-value	4e-31*	2e-4*	0.6	0.6	0.8	5e-11*
Cognitively unimpaired Aβ-negative participants (n=197)<br>R-squared =0.371						
β-coef	0.705	-0.212	0.104	0.098	-0.029	-0.348
P-value	3e-13*	0.002*	0.08	0.4	0.8	6e-5*

Supplementary Table 2: Cluster 11 consisted of proteins that are more highly expressed in the brain compared to the other highly expressed CSF proteins. Row 1: the proportion that had Medium/High level of detection in cerebral cortex. Rows 2 and 3: the normalized Transcripts Per Million [nTPM] in cerebral cortex/brain, comparing proteins in cluster 11 (n=219) to the rest of the highly detectable proteins (n=1512). n_{tot} is the number of proteins that existed in that dataset. Statistical difference was assessed with two-sided Mann-Whitney U test.

	**Cluster 11 (n=219)**	**Other proteins (n=1512)**	**Mann-Whitney U test result**
Normal tissue data<br>Tissue: cerebral cortex<br>Unit: [Medium/High]	n_{tot}: 180<br>$n_{elevated}$: 131<br>73%	n_{tot}: 1109<br>$n_{elevated}$: 532<br>48%	
RNA consensus tissue gene data<br>Tissue: cerebral cortex<br>Unit: [nTPM]	n_{tot}: 217<br>mean: 73.3<br>CI: (0.8,206)	n_{tot}: 1494<br>mean: 48.8<br>CI: (0,154)	P-value = 7e-14

RNA single cell type tissue cluster data<br>Tissue: brain<br>Unit: [nTPM]	n_{tot}: 216<br>mean: 93.5<br>CI: (0,442)	n_{tot}: 1494<br>mean: 36.6<br>CI: (0,112)	P-value = 1e-13

Supplementary Table 3: Performance details for all models with and without references. Quantitative details for the three models evaluated on BF2 test dataset and independent cohort BF1, as presented in Fig. 5. AUCs were compared with one-sided ROC test using bootstrapping (n_{iter} = 2,000). P-values were adjusted for multiple comparisons by Benjamini–Hochberg method. All models were adjusted for age and sex. Exact P-values are reported inside parenthesis, and for convenience, they are also denoted with * when $P < 0.05$ and ** when $P < 0.01$.

	**AUC (95% CI)**	**AUC difference vs. no reference (P-value)**	**AUC difference vs. mean CSF (P-value)**
P-tau181→TauPET (BF2)			
No reference	0.828 (0.750, 0.906)	-	-
Aβ40	0.933 (0.896, 0.970)	0.105 (0.002**)	0.0386 (0.01*)
Mean CSF level	0.895 (0.847, 0.943)	0.0663 (0.009**)	-
NTRK3	0.920 (0.879, 0.962)	0.0981 (0.003**)	0.0255 (0.01*)
NTRK2	0.923 (0.882, 0.964)	0.0947 (0.002**)	0.0284 (0.01*)
BLMH	0.908 (0.861, 0.954)	0.0794 (0.005**)	0.0131 (0.2)
SVD1 (NTRK3, NTRK2 and BLMH)	0.928 (0.889, 0.967)	0.0995 (0.002**)	0.0332 (0.01*)
CBLN4	0.944 (0.907, 0.981)	0.115 (0.002**)	0.0491 (0.01*)
PTPRN2	0.937 (0.901, 0.974)	0.109 (0.002**)	0.0427 (0.01*)
PTPRS	0.928 (0.889, 0.967)	0.0997 (0.003**)	0.0334 (0.01*)

SVD2 (CBLN4, PTPRN2 and PTPRS)	0.946 (0.913, 0.980)	0.118 (0.002**)	0.0518 (0.009**)
Aβ42→AβPET (BF2)			
Baseline	0.966 (0.939, 0.993)	-	-
Aβ40	0.992 (0.982, 1)	0.0261 (0.03*)	0.0148 (0.1)
Mean CSF level	0.977 (0.956, 0.998)	0.0113 (0.04*)	-
NTRK3	0.983 (0.966, 1)	0.0169 (0.03*)	0.00563 (0.2)
NTRK2	0.987 (0.972, 1)	0.0208 (0.03*)	0.00951 (0.1)
BLMH	0.986 (0.971, 1)	0.0201 (0.03*)	0.00880 (0.1)
SVD1	0.987 (0.972, 1)	0.0208 (0.03*)	0.00951 (0.1)
CBLN4	0.983 (0.966,1)	0.0176 (0.03*)	0.00634 (0.2)
PTPRN2	0.982 (0.965,1)	0.0162 (0.03*)	0.00493 (0.2)
PTPRS	0.983 (0.967,1)	0.0176 (0.03*)	0.00634 (0.1)
SVD2 (CBLN4, PTPRN2 and PTPRS)	0.985 (0.969, 1)	0.0190 (0.03*)	0.00775 (0.1)
P-tau181→ADDconv (BF2)			
No reference	0.866 (0.767,0.950)	-	-
Aβ40	0.898 (0.814, 0.965)	0.0324 (0.2)	-0.00629 (0.5)
Mean CSF level	0.905 (0.821, 0.970)	0.0394 (0.1)	-
NTRK3	0.920 (0.856, 0.971)	0.0544 (0.1)	0.0157 (0.4)
NTRK2	0.912 (0.842, 0.967)	0.0459 (0.2)	0.00722 (0.4)
BLMH	0.911 (0.843, 0.963)	0.0447 (0.1)	0.00595 (0.4)
SVD1 (NTRK3, NTRK2 and BLMH)	0.918 (0.855,0.970)	0.0524 (0.1)	0.0142 (0.4)
CBLN4	0.925 (0.866, 0.971)	0.0595 (0.1)	0.0208 (0.4)

PTPRN2	0.925 (0.866, 0.974)	0.0593 (0.1)	0.0206 (0.4)
PTPRS	0.922 (0.859, 0.975)	0.0561 (0.1)	0.0173 (0.4)
SVD2 (CBLN4, PTPRN2 and PTPRS)	0.930 (0.870, 0.976)	0.0639 (0.1)	0.0258 (0.4)
P-tau181→ADDconv (BF1)			
No reference	0.880 (0.838, 0.920)	-	-
Aβ40	0.933 (0.900, 0.962)	0.0538 (0.02*)	0.0259 (0.1)
Mean CSF level	0.908 (0.870, 0.946)	0.0278 (0.06)	-
NTRK3	0.935 (0.906, 0.961)	0.0546 (0.009**)	0.266 (0.03*)
NTRK2	0.932 (0.901, 0.960)	0.0523 (0.009**)	0.0248 (0.03*)
BLMH	0.896 (0.854, 0.937)	0.0169 (0.1)	-0.0112 (0.8)
SVD1	0.926 (0.888, 0.958)	0.0500 (0.03*)	0.0185 (0.1)
Aβ42→AβPET (BF1)			
No reference	0.916 (0.867, 0.962)	-	-
Aβ40	0.970 (0.938, 0.996)	0.0538 (0.006**)	0.0455 (0.02*)
Mean CSF level	0.924 (0.877, 0.968)	0.00879 (0.2)	-
NTRK3	0.932 (885, 0.974)	0.0162 (0.2)	0.00791 (0.2)
NTRK2	0.931 (0.884, 0.971)	0.0154 (0.2)	0.00713 (0.2)
BLMH	0.920 (0.870, 0.965)	0.00458 (0.3)	-0.00371 (0.6)
SVD1	0.933 (0.883, 0.975)	0.0167 (0.2)	0.00820 (0.2)

Supplementary Table 4: Effect sizes of association with CSF P-tau181 for sTREM2, sAXL, sTyro3 and YKL-40 without and with reference proteins. The proteins were evaluated in linear regression models with CSF P-tau181 as outcome. All models were adjusted for age and sex. The change in β-coefficients, P-values (two-sided) and R-squared when also adjusting for a reference protein (Aβ40, NTRK3 or CBLN4) was the measures of interest. All proteins were highly associated with CSF P-tau181 when not using a reference protein, but when adding a reference, the effect size decreased severely/disappeared. All models included NC (n=172), SCD (n=92) and MCI (n=145) participants from BF2. P-values were not adjusted for multiple comparisons.

		Without reference	With reference Aβ40		With reference NTRK3		With reference CBLN4	
		**Protein**	**Protein**	**Aβ40**	**Protein**	**NTRK3**	**Protein**	**CBLN4**
**Protein: sTREM2**	β-coef	0.346	0.102	0.569	0.0658	0.495	0.131	0.480
	P-value	1e-11	0.03	5e-33	0.1	6e-22	6e-3	9e-26
	R-squared	0.20	0.44		0.37		0.40	
**Protein: sAXL**	β-coef	0.488	0.174	0.476	0.176	0.385	0.186	0.394
	P-value	1e-26	7e-4	2e-19	0.01	7e-8	2e-3	4e-11
	R-squared	0.33	0.45		0.37		0.40	
**Protein: sTyro3**	β-coef	0.47	0.113	0.515	0.0307	0.502	0.136	0.435
	P-value	4e-23	0.03	2e-21	0.7	4e-10	0.02	9e-14
	R-squared	0.30	0.44		0.36		0.39	
**Protein: YKL-40**	β-coef	0.567	0.329	0.470	0.350	0.384	0.350	0.398
	P-value	5e-26	8e-11	3e-27	1e-10	2e-16	2e-11	1e-19
	R-squared	0.32	0.49		0.43		0.45	

Supplementary Table 5: Effect sizes of association with CSF P-tau181 for α-synuclein without and with reference proteins. α-synuclein was evaluated in linear regression models with CSF P-tau181 as outcome. All models were adjusted for age and sex. The change in β-coefficients, P-values (two-sided) and R-squared when also adjusting for a reference protein (Aβ40, NTRK3 or CBLN4) was the measures of interest. α-synuclein was highly associated with CSF P-tau181 when not using a reference protein, but when adding a reference in the model the association decreases. All models included AD dementia participants (n=210) from BF2. P-values were not adjusted for multiple comparisons.

		**Without reference**	**With reference Aβ40**		**With reference NTRK3**		**With reference CBLN4**	
		**Protein**	**Protein**	**Aβ40**	**Protein**	**NTRK3**	**Protein**	**CBLN4**
**Protein: α-synuclein**	β-coef	0.37	0.213	0.589	0.222	0.484	0.207	0.541
	P-value	8e-8	2e-4	3e-20	3e-4	1e-12	4e-4	9e-17
	R-squared	0.19	0.48		0.38		0.44	

Supplementary Table 6: Effect sizes of *APOE* ε4 allele association with ApoE4 CSF protein expression without and with reference proteins. The number of *APOE* ε4 alleles is evaluated in linear regression models with the protein expression of CSF ApoE4 as outcome. All models were adjusted for age and sex. The change in β-coefficients, P-values (two-sided) and R-squared when also adjusting for a reference protein (Aβ40, NTRK3 or CBLN4) was the measures of interest. The alleles were encoded as follows: ε2ε4, ε3ε4 = 1 and ε4ε4=2. The BF1 dataset was used, only including participants with one or two ε4 alleles and complete measures for all other parameters (n=437). P-values were not adjusted for multiple comparisons.

		**Without reference**	**With reference Aβ40**		**With reference NTRK3**	
		***APOE* genotype**	***APOE* genotype**	**Aβ40**	***APOE* genotype**	**NTRK3**
**Outcome Protein: ApoE4**	β-coef	0.580	0.590	0.429	0.578	0.441
	P-value	3e-40	2e-45	1e-27	1e-51	4e-33
	R-squared	0.34	0.52		0.53	

Supplementary Table 7: The selected pQTL-analysis proteins and their association with the mean CSF level. Linear regression models adjusted for age and sex, describing each protein's association with the mean CSF level in the BF1 dataset. The higher the association, the more likely to benefit from adjusting for the individual reference level when used as a biomarker. P-values were two-sided and not adjusted for multiple comparisons.

Protein	Linear Regression Parameters
VEGFA	β = 0.82, p=1e-120
sVCAM1	β = 0.64, p=1e-41
PLXNB1	β = 0.82, p=1e-180
PRTG	β = 0.85, p=1e-210
TFF3	β = 0.77, p=1e-120

Supplementary Table 8: Effect of genotype (SNP) in associations with CSF proteins without and with reference proteins. The proteins were evaluated in linear regression models, with the CSF protein as outcome and SNP as main predictor. In all models, age, sex, dementia diagnosis and ten genetic principal components were adjusted for. The change in association when also adjusting for a reference protein (Aβ40 or NTRK3) was the measure of interest. The changes in β-coefficients, P-values (two-sided) and R-squared were used to compare the effect of genotype between different models. In general, the trans-pQTL associations of the GMNC-OSTN were severely weakened when adjusting for a reference protein. All models included BF1 participants (n=1445). P-values were not adjusted for multiple comparisons.

		**Without reference**	**With reference Aβ40**		**With reference NTRK3**	
		**SNP**	**SNP**	**Aβ40**	**SNP**	**NTRK3**
**Outcome Protein: VEGFA**<br><br>**SNP: rs57712768**	β-coef	0.15	0.094	0.63	0.020	0.86
	P-value	2e-8	6e-6	1e-145	0.1	1e-150
	R-squared	0.089	0.45		0.76	
**Outcome Protein: *sVCAM1***<br><br>**SNP: rs146550622**	β-coef	0.15	0.12	0.35	0.089	0.37
	P-value	8e-8	8e-6	1e-36	4e-4	2e-42
	R-squared	0.20	0.31		0.31	
	β-coef	0.16	0.080	0.71	0.027	0.86

**Outcome Protein:** ***PLXNB1*** **SNP: rs4687181**	P-value	2e-9	5e-5	4e-191	0.06	1e-150
	R-squared	0.054	0.52		0.72	
**Outcome Protein: PRTG** **SNP: rs71635338**	β-coef	0.16	0.11	0.52	0.022	0.89
	P-value	9e-10	3e-6	5e-88	0.07	1e-150
	R-squared	0.073	0.32		0.78	
**Outcome Protein: TFF3** **SNP: rs78054167**	β-coef	0.15	0.11	0.33	0.039	0.62
	P-value	2e-8	4e-6	1e-36	0.04	5e-164
	R-squared	0.16	0.26		0.49	

Supplementary Table 9: OLINK Panel statistics. Panel statistics of proteins with missing frequency > 50%.

OLINK Panel	Proteins with Missing Frequency > 50%
Explore 384 Cardiometabolic	19%
Explore 384 Cardiometabolic II	59%
Explore 384 Inflammation	43%
Explore 384 Inflammation II	42%
Explore 384 Neurology	35%
Explore 384 Neurology II	78%
Explore 384 Oncology	45%
Explore 384 Oncology II	84%

Supplementary Table 10: Association with the mean CSF level for the proteins that were analyzed. Linear regression models adjusted for age and sex, describing each protein's association with the mean CSF level in BF2 training dataset. Sorted in decreasing order. The higher the association, the more likely to benefit from adjusting for the individual reference level when used as a biomarker. P-values were two-sided and not adjusted for multiple comparisons.

Protein	Linear Regression Result
sAXL	β = 0.75, p=9e-150
sTyro3	β = 0.68, p=1e-105
sTREM2	β = 0.51, p=4e-46
YKL-40	β = 0.40, p=2e-24
GFAP	β = 0.38, p=5e-22
Neurogranin	β = 0.35, p=7e-24
T-tau	β = 0.34, p=2e-21
P-tau	β = 0.34, p=3e-21
S100	β = 0.29, p=1e-16
Aβ42	β = 0.24, p=1e-8
Alpha Synuclein	β = 0.23, p=8e-11
NfL	β = 0.10, p=0.007

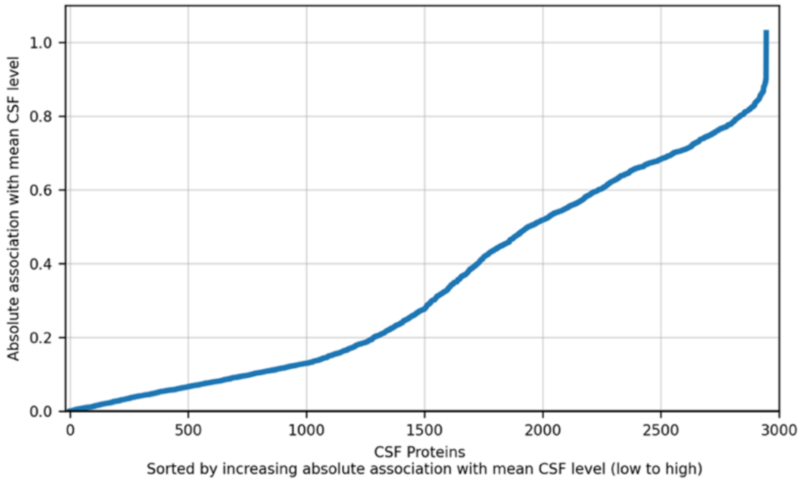

Supplementary Figure 1: **Absolute association (β-value) with mean CSF level for all 2944 proteins.** Computed in a linear regression model adjusted for age and sex with the BF2 training set (n=658). The result was used to sort the x-axis in Fig. 2 and Supplementary Fig. 3.

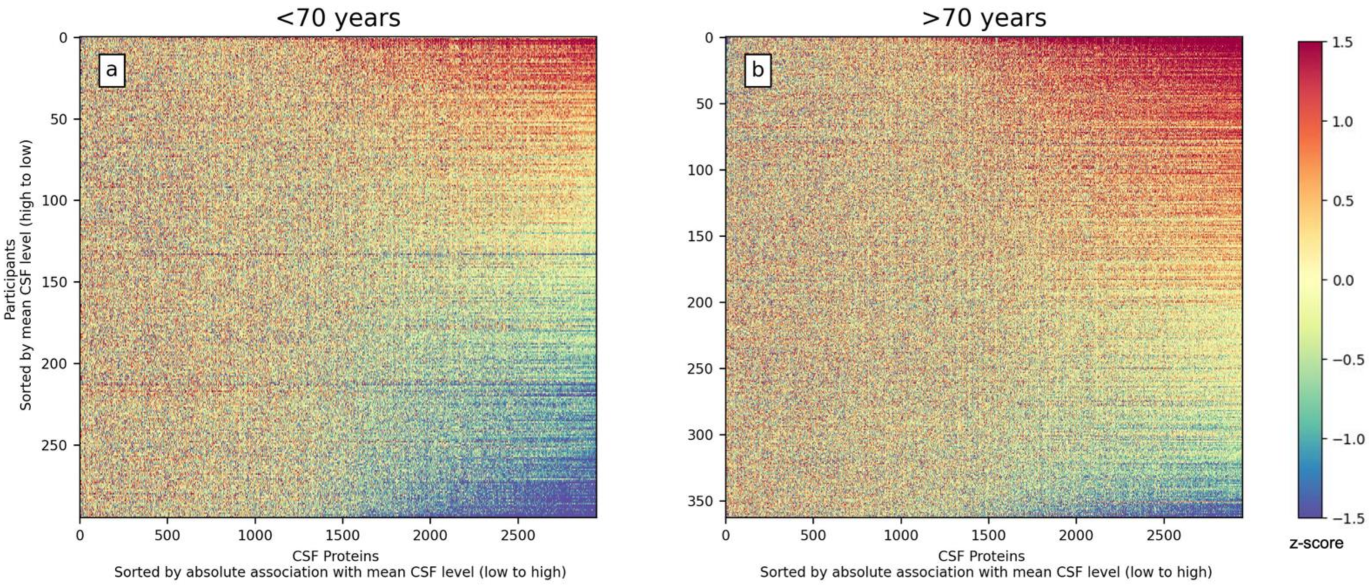

Supplementary Figure 2: Visualization of individual CSF levels stratified by age. As in Figure 2, for each participant (row) in the BF2 training set, the standardized concentration of 2,944 CSF proteins, sorted by increasing absolute association with the mean standardized CSF protein level is displayed. Systematic blue horizontal lines can be seen for individuals with consistently low values across most proteins, and correspondingly red horizontal lines for individuals with high values across most proteins (all relative to the total sample). a) below 70 years of age (n=295) and b) above 70 years of age (n=363).

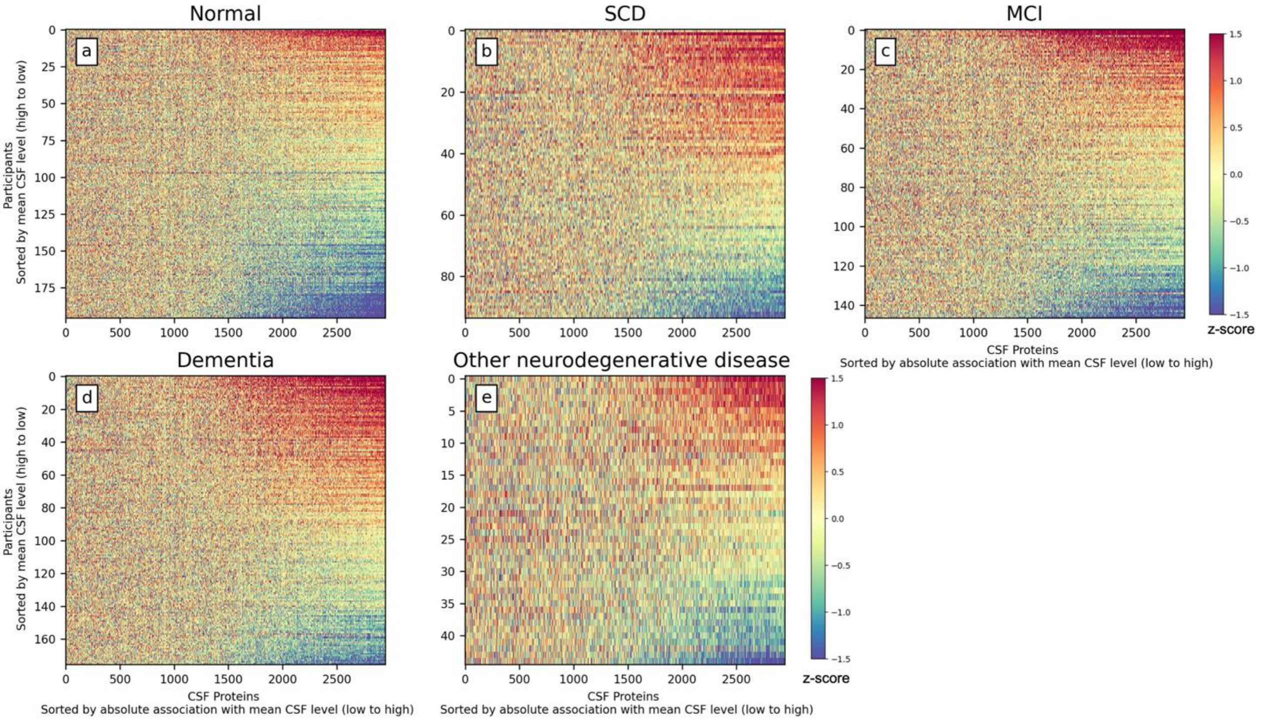

Supplementary Figure 3: Visualization of individual CSF levels stratified by clinical status. As in Figure 2, for each participant (row) in the BF2 training set, the standardized concentration of 2,944 CSF proteins, sorted by increasing absolute association with the mean CSF protein level is displayed. Systematic blue horizontal lines can be seen for individuals with consistently low values across most proteins, and correspondingly red horizontal lines for individuals with high values across most proteins (all relative to the total sample). a) NC (n=196) b) SCD (n=94) c) MCI (n=147) d) Dementia (n=176) e) Other neurodegenerative disease (n=45).

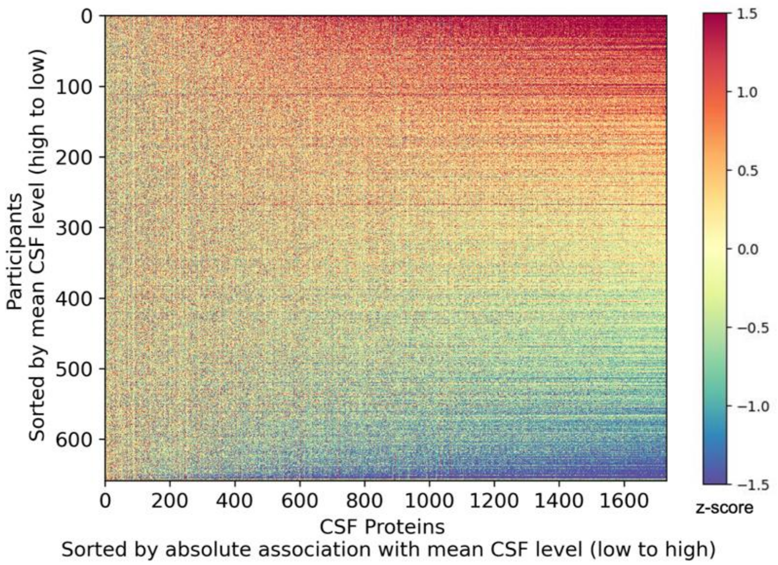

Supplementary Figure 4: Visualization of individual CSF levels only including proteins with missing frequency < 75%. For each participant (row) in the BF2 training set (n=658), the z-score of 1730 highly detected CSF proteins, sorted by absolute association with mean CSF level, is displayed. As expected, the subject-specific CSF level is prominently shown in highly expressed proteins.

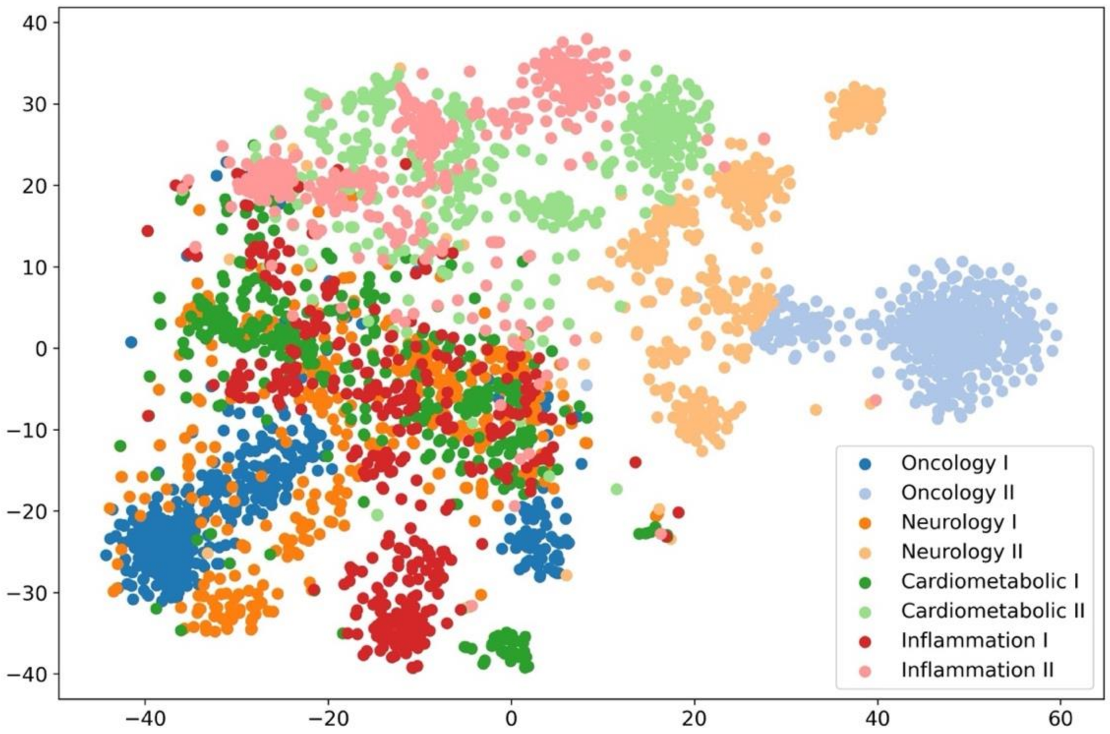

Supplementary Figure 5: **t-SNE OLINK panel coloring.** t-SNE dimensionality reduction of 658-dimensional space of participants into a two-dimensional space, with the 2943 OLINK proteins colored by their OLINK panel inclusion. Source data are provided as a Source Data file.

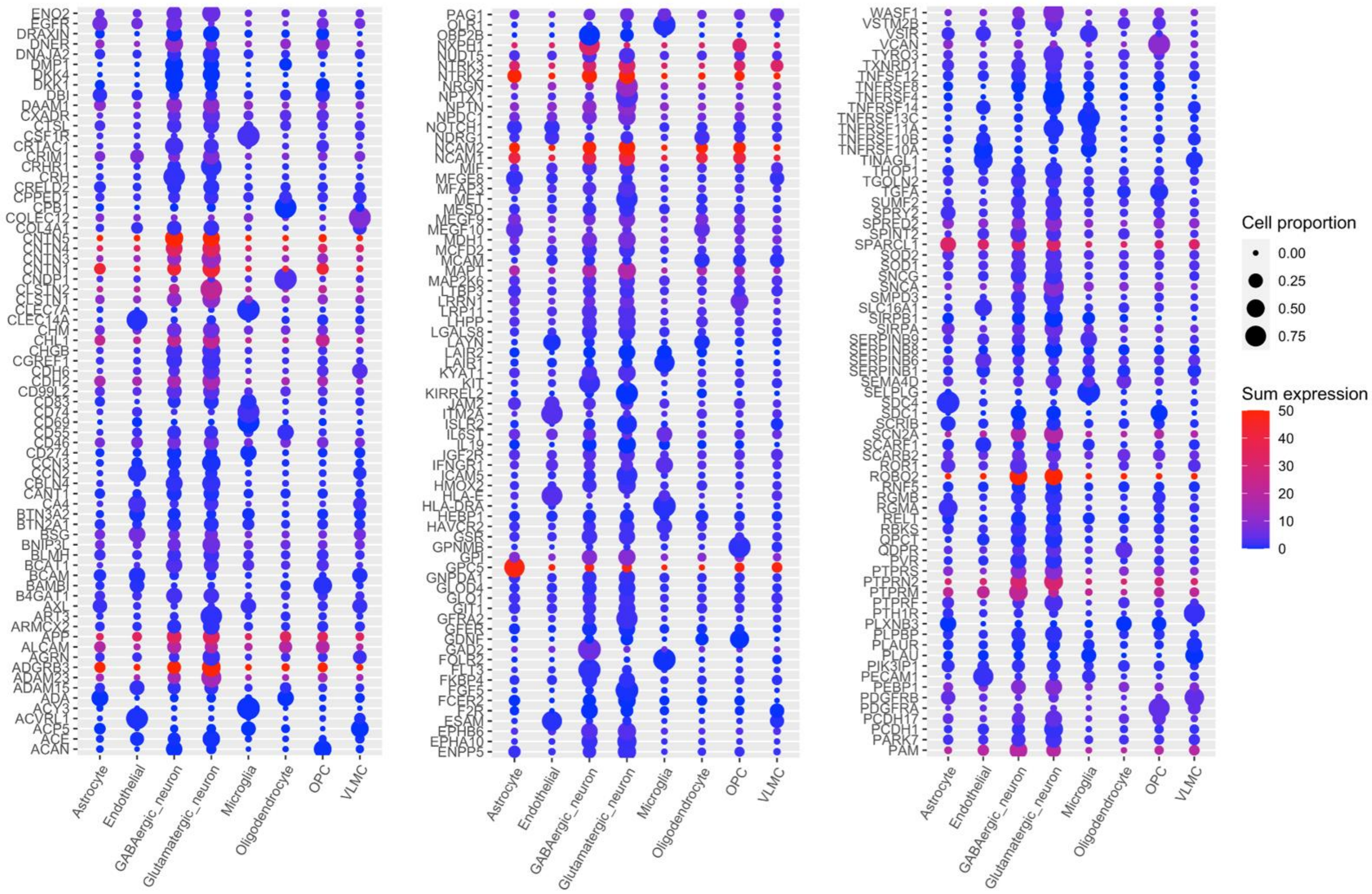

Supplementary Figure 6: **Cluster 11 cell type expression.** Average cell type expression profiles for 217 of the 219 genes corresponding to the proteins in cluster 11. Based on data from 5 post-mortem human brain specimens in the Allen Brain Data. Abbreviations: gamma-aminobutyric acid (GABA), Oligodendrocyte progenitor cell (OPC), vascular and leptomeningeal cell (VLMC).

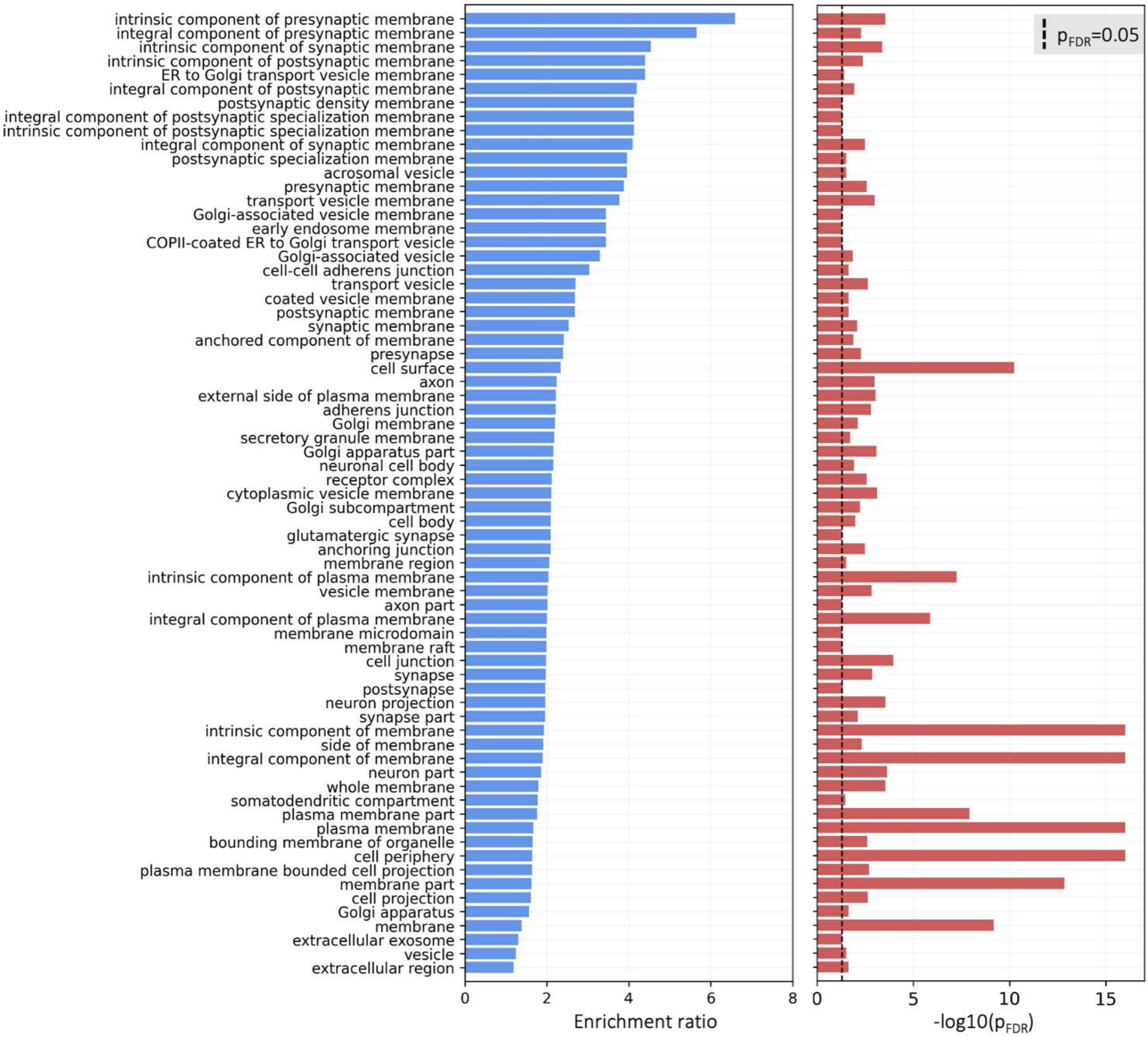

Supplementary Figure 7: **Cluster 11 cellular component enrichment analysis.** Over representation cellular component enrichment analysis for 217 of the 219 proteins in cluster 11 performed on the WEB-based Gene SeT AnaLysis Toolkit (WebGestalt). The background set was defined as all 2943 OLINK proteins.

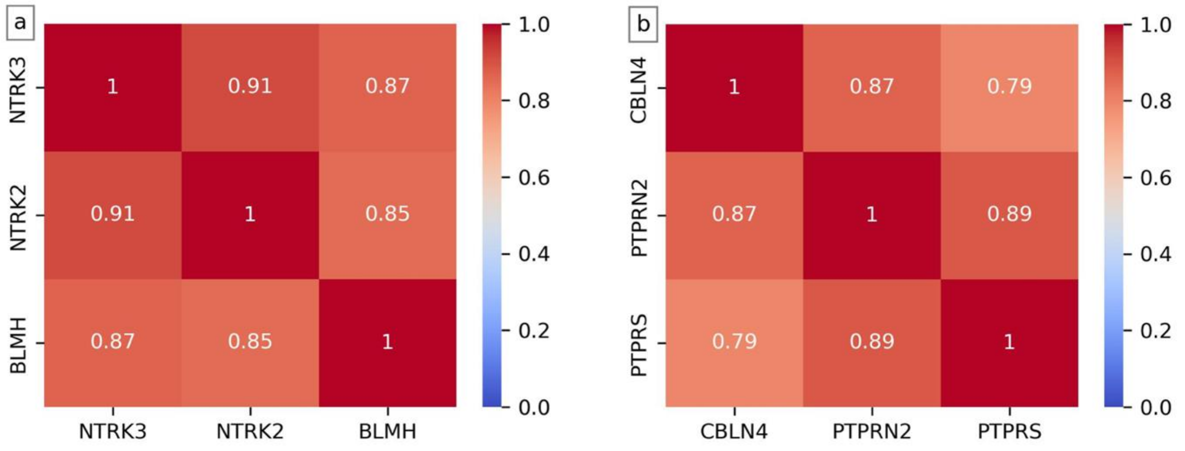

Supplementary Figure 8: Correlation matrix of general candidates. Pearson correlation matrix of the **a)** general reference protein candidates NTRK3, NTRK2 and BLMH and **b)** P-tau181 specific reference protein candidates CBLN4, PTPRN2 and PTPRS. Calculated in the BF2 training set (n=658).

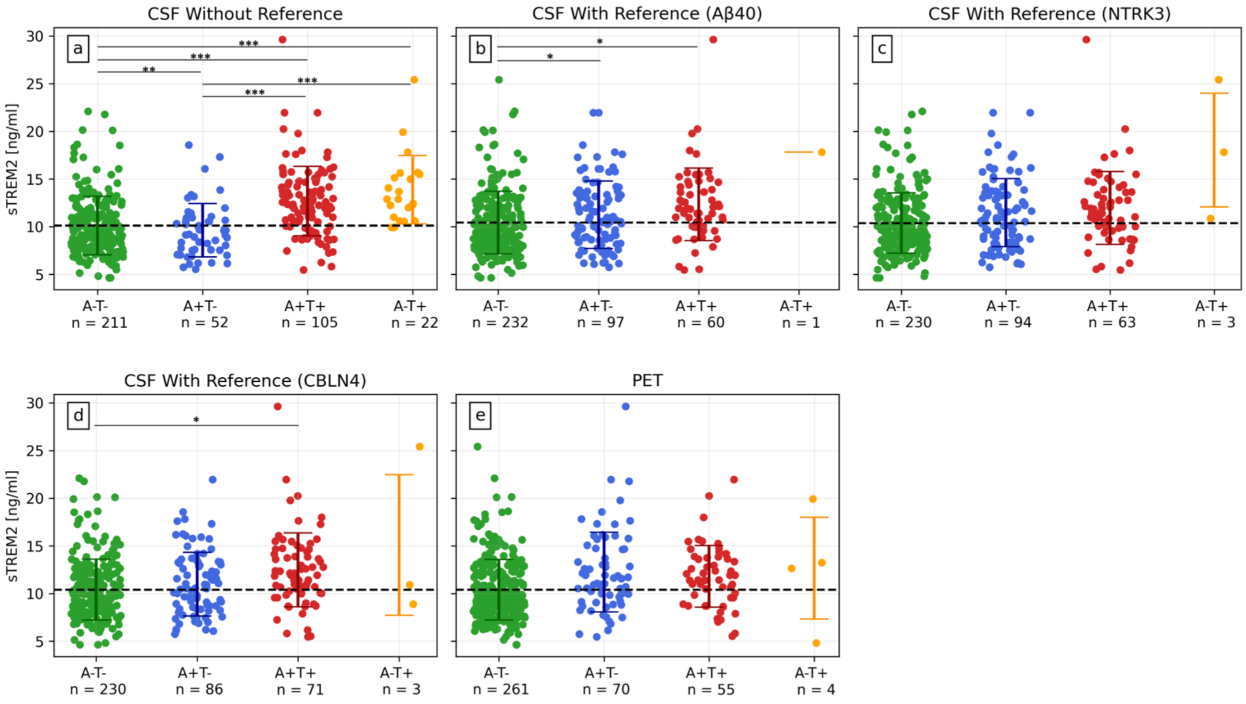

Supplementary Figure 9: sTREM2 levels by AT(N) grouping for NC, SCD and MCI. a) CSF grouping without reference. **b)** CSF grouping with Aβ40 as reference. **c)** CSF grouping with NTRK3 as reference. **d)** CSF grouping with CBLN4 as reference. **e)** PET grouping. The differences between groups were affected by how the grouping was performed. If using CSF grouping, adjusting for a reference protein reduced the group differences, creating a better concordance between CSF and PET grouping. P-values (adjusted for multiple comparisons) were assessed by a one-way ANCOVA adjusted for age and sex. Error bars represent the mean concentration ± one standard deviation. Source data are provided as a Source Data file.

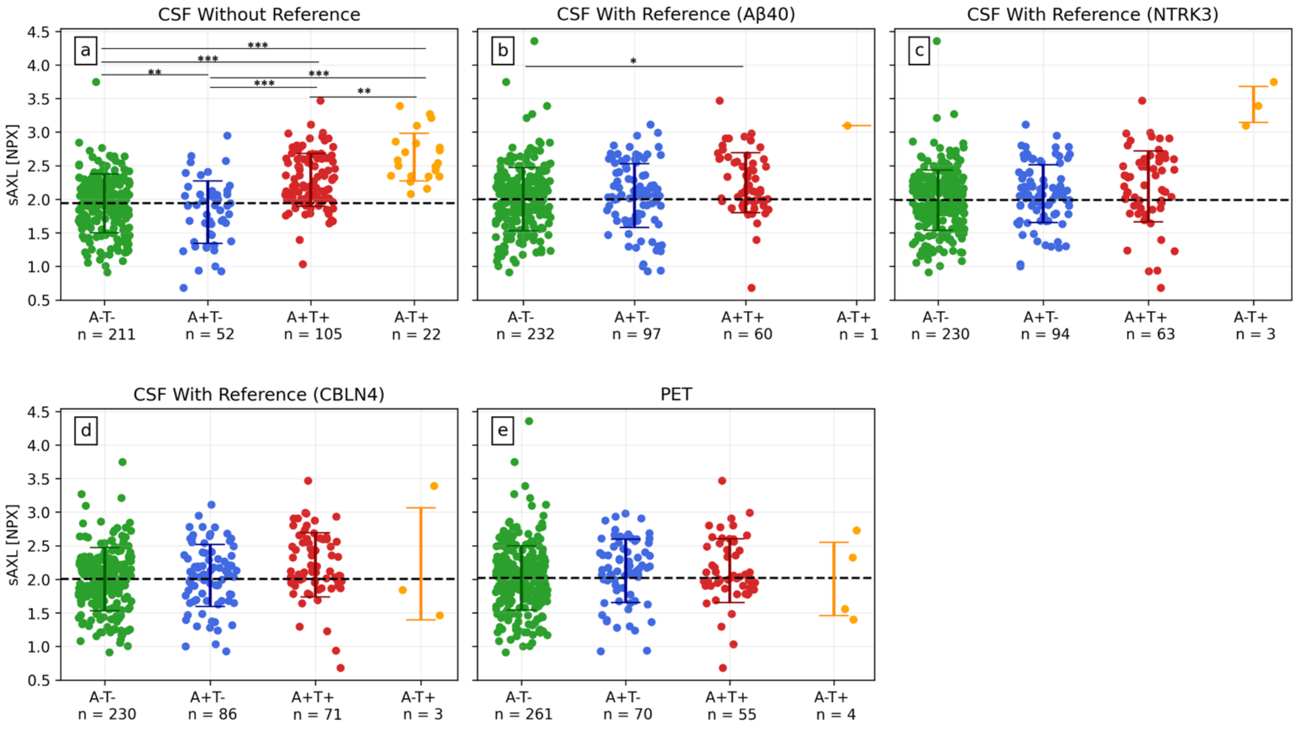

Supplementary Figure 10: sAXL levels by AT(N) grouping for NC, SCD and MCI. a) CSF grouping without reference. **b)** CSF grouping with Aβ40 as reference. **c)** CSF grouping with NTRK3 as reference. **d)** CSF grouping with CBLN4 as reference. **e)** PET grouping. The differences between groups were affected by how the grouping was performed. If using CSF grouping, adjusting for a reference protein reduced the group differences, creating a better concordance between CSF and PET grouping. *P*-values (adjusted for multiple comparisons) were assessed by a one-way ANCOVA adjusted for age and sex. Error bars represent the mean concentration ± one standard deviation. Source data are provided as a Source Data file.

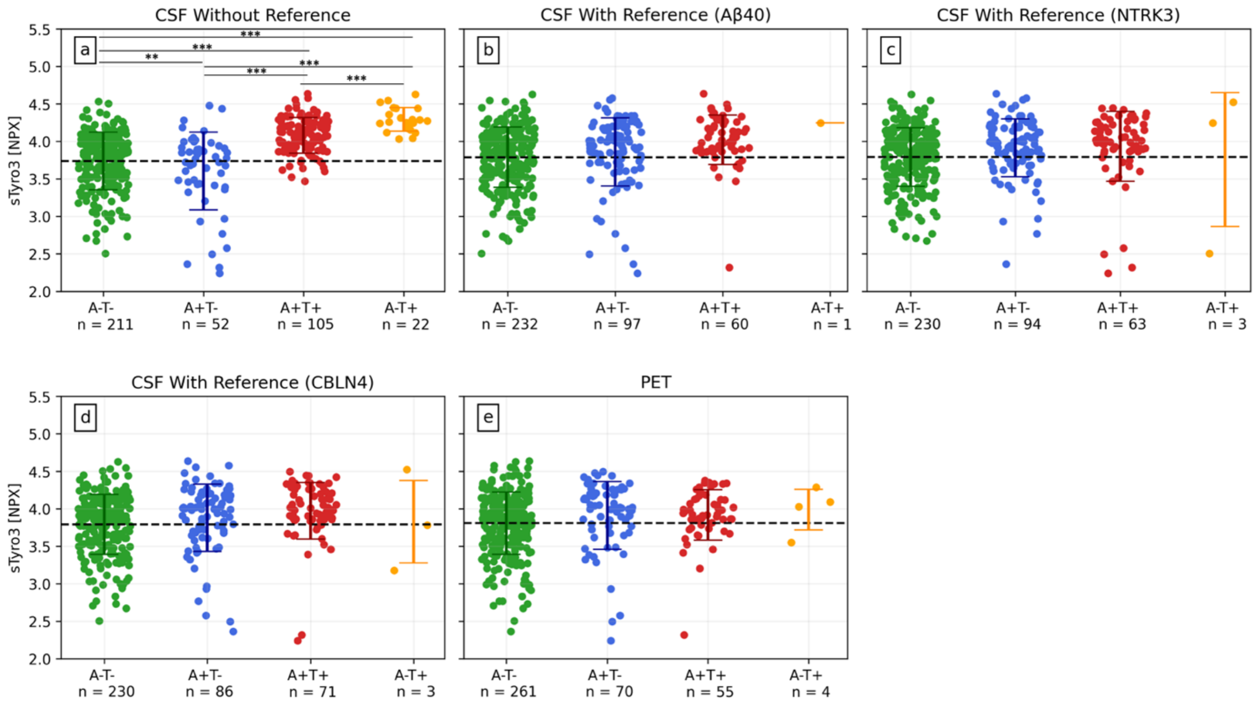

Supplementary Figure 11: sTyro3 levels by AT(N) grouping for NC, SCD and MCI. a) CSF grouping without reference. **b)** CSF grouping with Aβ40 as reference. **c)** CSF grouping with NTRK3 as reference. **d)** CSF grouping with CBLN4 as reference. **e)** PET grouping. The differences between groups were affected by how the grouping was performed. If using CSF grouping, adjusting for a reference protein reduced the group differences, creating a better concordance between CSF and PET grouping. *P*-values (adjusted for multiple comparisons) were assessed by a one-way ANCOVA adjusted for age and sex. Error bars represent the mean concentration ± one standard deviation. Source data are provided as a Source Data file.

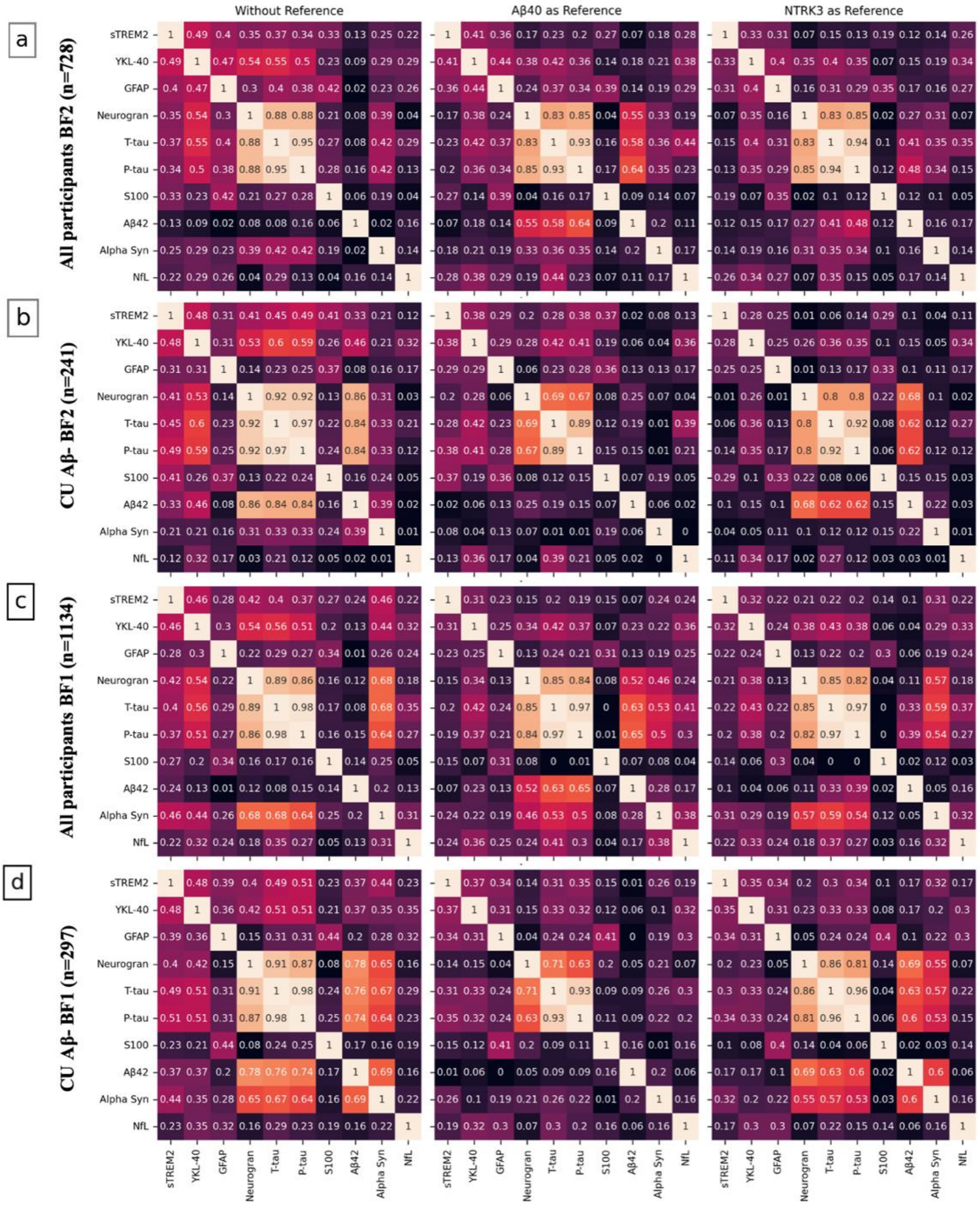

Supplementary Figure 12: Correlation matrices for ten NeuroToolKit proteins in BF1 and BF2 with and without adjusting for a reference protein. Partial correlation matrices for ten NeuroToolKit proteins in BF2 training and BF1 datasets, adjusting for age and sex. Proteins were sorted according to decreasing association with mean CSF level (se Supplementary Tab. 10). From these results, a comparison of the change in correlation when also adjusting for a reference protein (Aβ40 or NTRK3) can be made. Almost all correlations were severely reduced when adjusting for a reference protein, particularly evident in cognitively unimpaired individuals without AD pathology. **a)** All participants in BF2 (n=728). **b)** Cognitively unimpaired Aβ-negative BF2 participants (n=241). **c)** All participants in BF1 (n=1134). **d)** Cognitively unimpaired Aβ-negative BF1 participants (n=297).

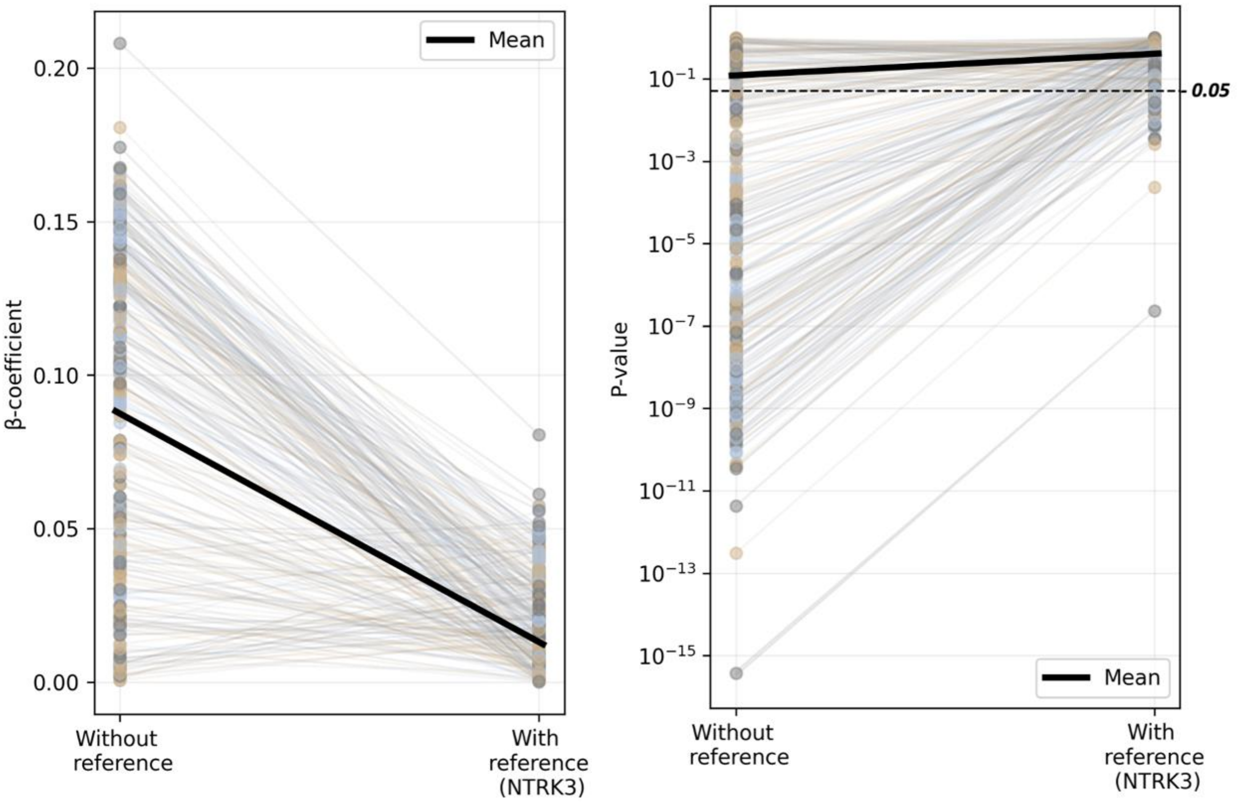

Supplementary Figure 13: Effects of the most common CSF *trans*-pQTL variant rs71635338 in associations with CSF proteins without and with reference protein NTRK3. The SNP rs71635338 is evaluated by association to each CSF OLINK protein in BF1 (n=367, all except NTRK3) in a linear regression model, without and with adjustment for the reference protein NTRK3. The difference is shown by β-coefficient (left) and two-sided P-value (right) for every protein. On average, β-coefficients decreased from 0.088 to 0.012, and P-values increased from 0.12 to 0.41 when adjusting for a reference protein. All models included BF1 participants (n=1445) and were adjusted for age, sex, dementia diagnosis and ten genetic principal components. A variance inflation factors (VIF) analysis revealed that the models did not violate collinearity assumptions, as 96% av the proteins had VIF <5. Source data are provided as a Source Data file.

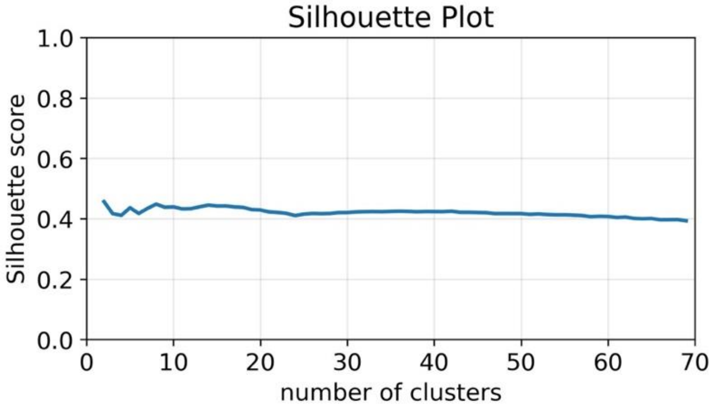

Supplementary Figure 14: Mean silhouette score for K-means clustering of t-SNE space. For each K, a score was computed as the average of 20 different random initializations. As seen here, the silhouette score did not vary much between different Ks in the range 2-70, hence not evidently favoring any specific K.

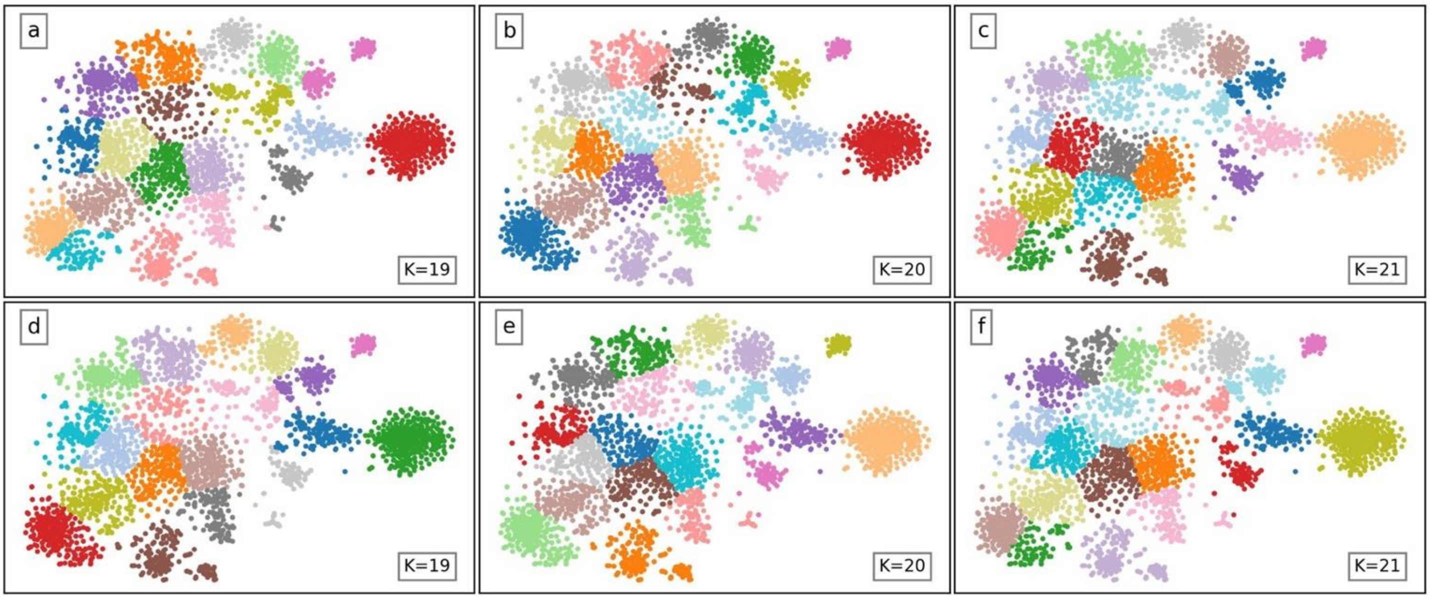

Supplementary Figure 15: Semi-supervised K-means clustering of t-SNE space for different Ks and random initialization seeds. t-SNE dimensionality reduction of 658-dimensional space of participants into a two-dimensional space, with the 2944 CSF proteins presented as scatter points colored by different K-means clusterings. As seen in the six subfigures, some variations in the result are given when changing K and random initialization seeds. The semi-supervised K-means clustering with K=20 is a non-unique nor mathematically optimized tool to select a subset of proteins. Still, it fulfills the purpose of adding robustness when analyzing expression characteristics rather than single proteins in a reproducible way. This could also have been achieved with different Ks and random initialization seeds as seen in the figure, making the results highly reproducible.

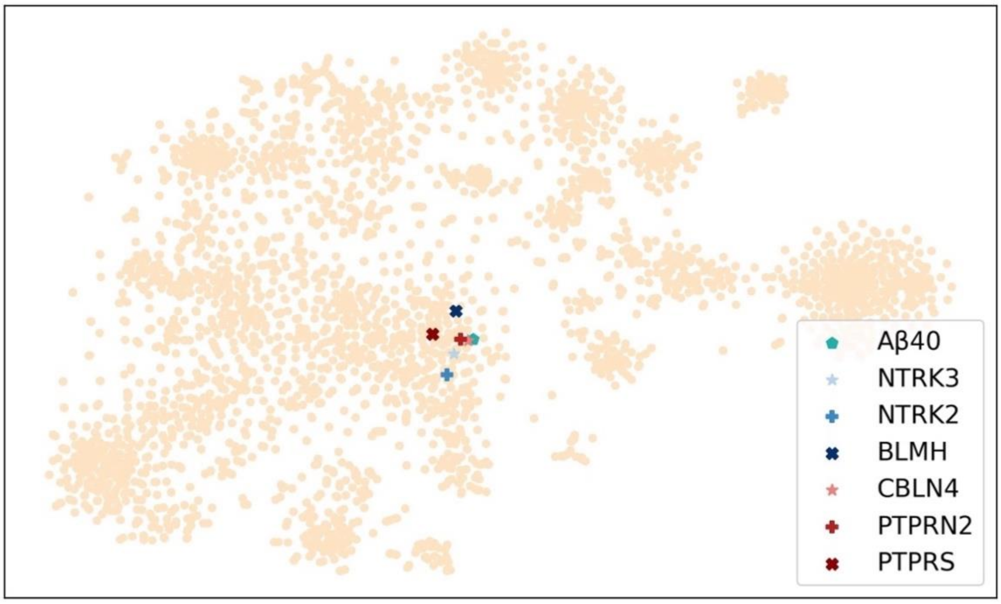

Supplementary Figure 16: Location of the examined reference protein candidates in t-SNE map. t-SNE dimensionality reduction of 658-dimensional space of participants into a two-dimensional space, with the 2944 CSF proteins presented as scatter points. The examined reference protein candidates are marked out in t-SNE space. As seen, the area of interest is covered by single clusters in all examples in Supplementary Fig. 15, indicating that the clustering could be performed with different Ks and random initialization seeds and still include these candidates.

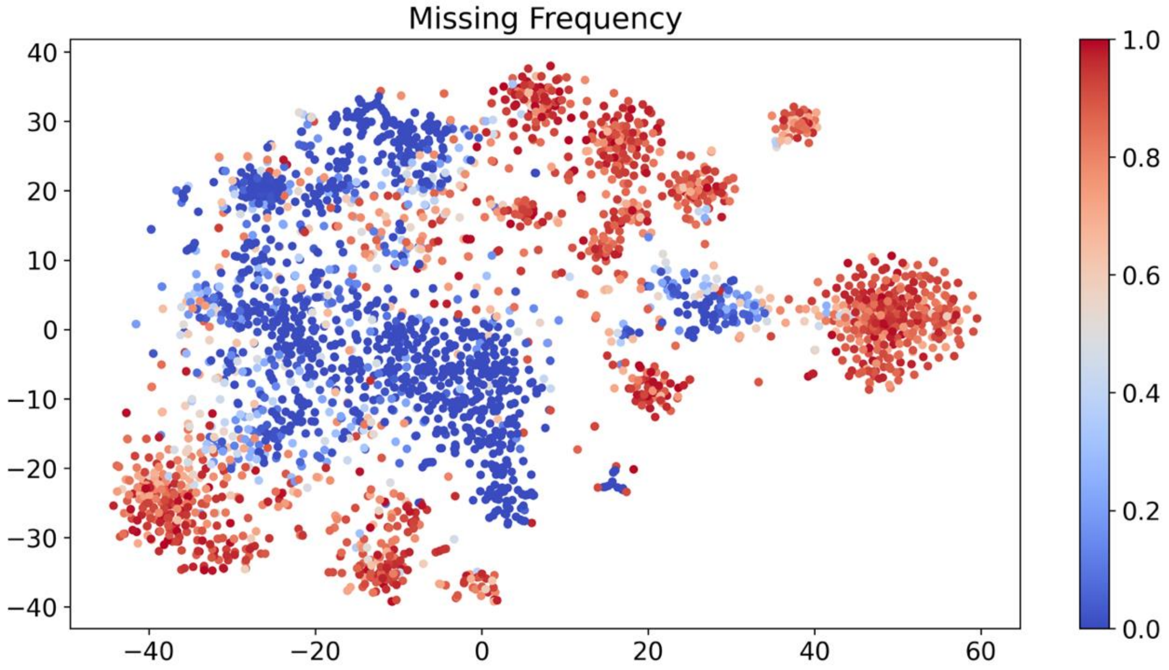

Supplementary Figure 17: Missing frequency t-SNE map. t-SNE dimensionality reduction of 658-dimensional space of participants into a two-dimensional space, with the 2944 CSF proteins presented as scatter points colored according to missing frequency for each protein.

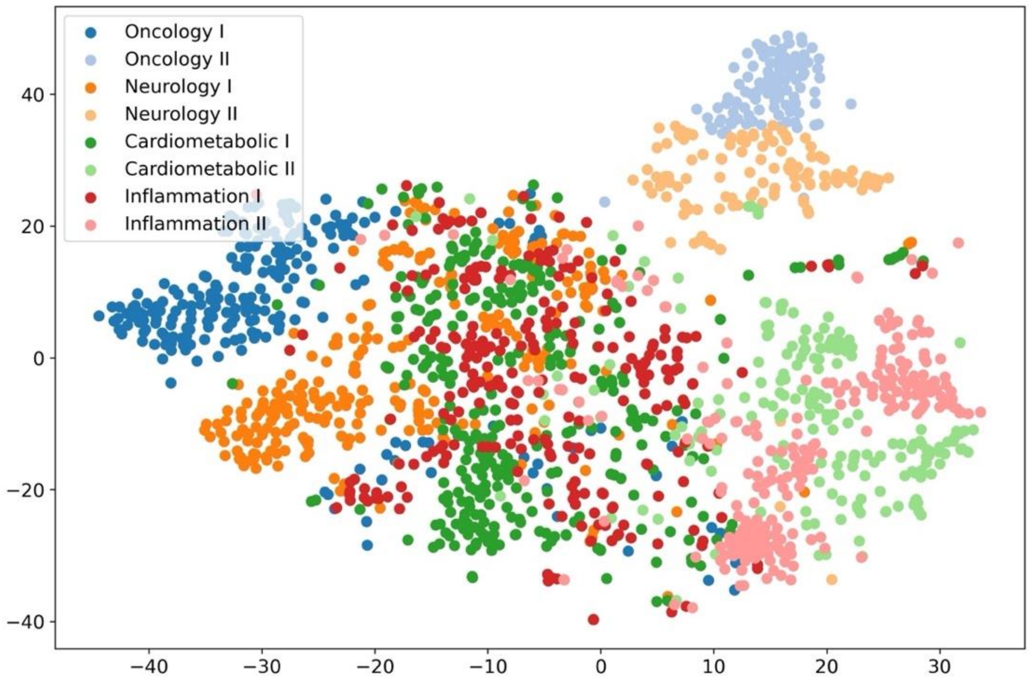

Supplementary Figure 18: t-SNE on reduced data colored by panel. t-SNE dimensionality reduction from BF2 training set (n=658) with proteins of missing frequency < 75% ($n_{proteins}$=1,730), colored by OLINK panel. As seen, the panel division is like that of the full dataset (see Supplementary Fig. 4), where Oncology II and Neurology II are separated but close to each other, majority of Oncology I and Neurology I next to each other, and Cardiometabolic II and Inflammation II together.

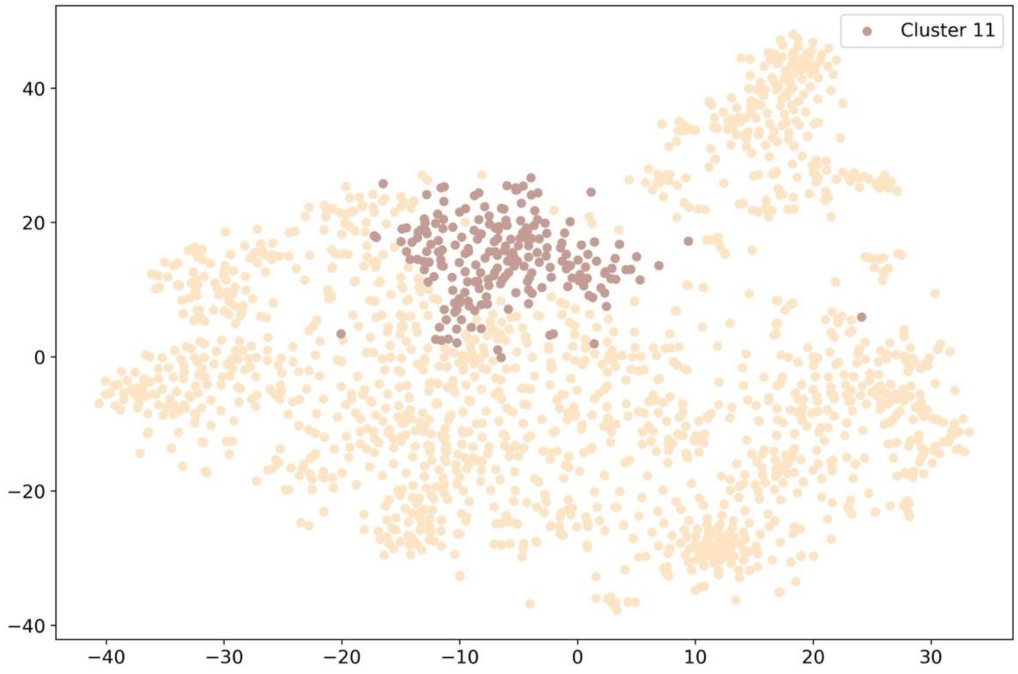

Supplementary Figure 19: t-SNE on reduced data highlighting cluster 11. t-SNE dimensionality reduction from BF2 training set (n=658) with proteins of missing frequency < 75% ($n_{proteins}$=1,730) highlighting cluster 11. The proteins of the cluster are still located close to each other, covering a central area of the map.

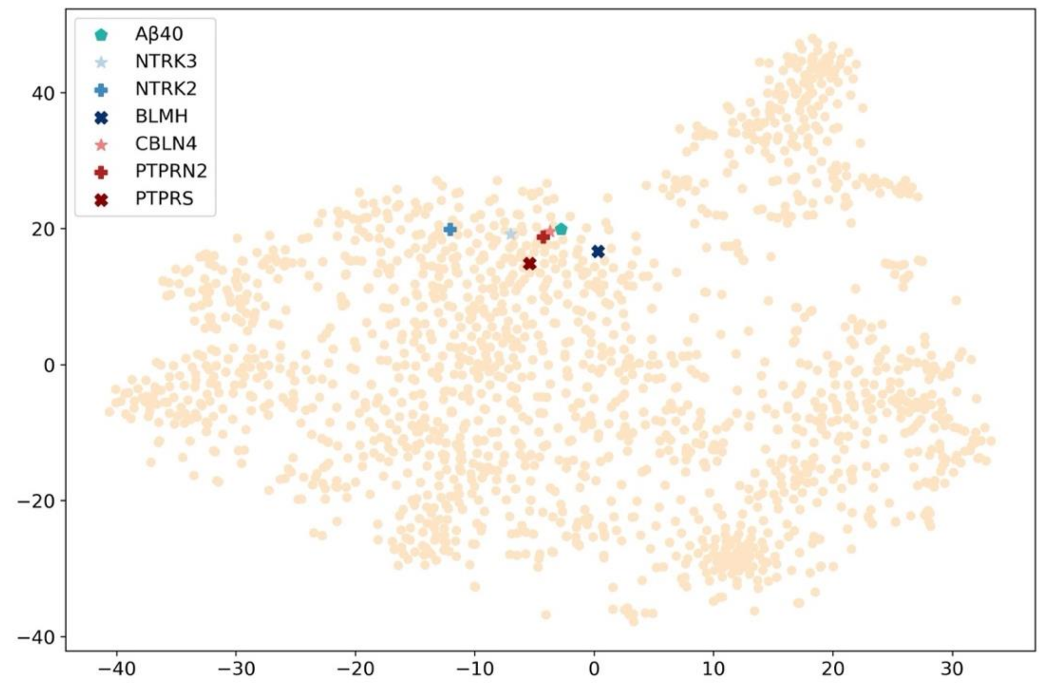

Supplementary Figure 20: t-SNE on reduced data highlighting reference protein candidates. t-SNE dimensionality reduction from BF2 training set (n=658) with proteins of missing frequency < 75% ($n_{proteins}$=1,730), highlighting the suggested reference protein candidates. For this reduced dataset, the candidates are still located closely together.

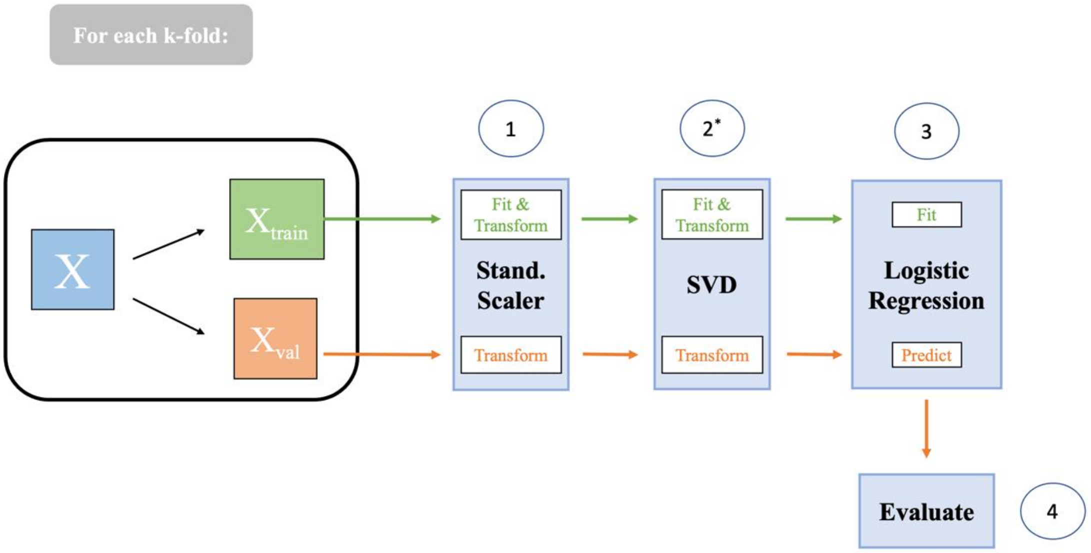

Supplementary Figure 21: **Pipeline for pre-processing steps of all models.** All fits (standard scaling, SVD and logistic regression) were applied only to training data (either training dataset or training folds/samples in 10-fold-cross-validation/bootstrap). Both training and validation data were then transformed accordingly. Test data was treated as validation data in this pipeline. Step 2 was skipped unless specified otherwise.

* SVD was only applied when a singular value decomposition was used as reference.

Supplementary Methods

OLINK Data Handling

Quality Control

Each OLINK sample has gone through three internal and three external quality control steps. The internal controls (Incubation, Extension and Amplification control) are used to monitor quality of assay performance and individual samples, as well as generation of NPX values. The external controls are used for data normalization, to assess variation between runs and plates, to calculate limit of detection (LOD) and discover contamination. All samples are included in the data output file but labeled with WARN if they do not pass a certain quality control. 95% of all datapoints passed QC for BF2. All data was kept for the analysis to maintain statistical power, as the existing QC/assay warnings were distributed between different proteins and participants.

Limit of Detection

Limit of Detection (LOD) is calculated from the background plus three standard deviations (assay specific), estimated from negative controls. LODs are computed separately for each assay and plate. Due to the S-curve relationship of OLINK platform data, concentrations below LOD are at risk of being non-linear (meaning that 1 NPX differences do not correspond to 2x change). This may bias results as values tend to be condensed to a small range. Still, the data can contain informative structures and can differ between groups, which is why OLINK do not recommend removing data points below LOD. No LOD filtering was therefore performed in this work. The percentage of proteins below LOD are referred to as the missing frequency. For the BF2 CSF OLINK data, the within-panel statistics of number of proteins with missing frequency > 50% can be seen in Supplementary Tab. 9.

LOD Sensitivity Analysis

To create Fig. 2 and 3, all 2,943 OLINK proteins were included. To estimate the effect of low LODs, the t-SNE map was colored according to missing frequency in Supplementary Fig. 17. As seen, the area of highly

detected proteins (blue) overlaps with the area of proteins strongly associated with the mean CSF level (see Fig. 3c). Additionally, the most well-separated clusters tend to contain a high extent of proteins below LOD. As there still exist clear clustering characteristics for proteins with high missing frequency, both within and between panels (compare with Supplementary Fig. 5), the data evidently contains identifying structures. We therefore considered it reasonable to include all the proteins in this analysis. To ensure these qualities did not affect conclusions about cluster 11, a sensitivity analysis of the visualization of concept and t-SNE dimensionality reduction was performed, filtering out all proteins with missing frequency > 75%. When doing so, 1,730 proteins maintained included. The result can be seen in Supplementary Fig. 18-20 and are in line with the findings from using all 2,943 OLINK proteins.

K-means Robustness Analysis

The K-means robustness analysis aims to provide evidence that the results were not heavily dependent on the selection of K or random initialization seed. A silhouette plot is provided (Supplementary Fig. 14), showing how selecting a certain K is neither obvious nor of great importance for generating similar results. The silhouette score is a metric that evaluates the quality of a clustering result, considering cluster wise intra- and inter-distances. The score ranges between -1 and 1, where 1 represents a dense and well-separated clustering, 0 overlapping clusters with samples nearby decision boundaries and negative values that samples have likely been assigned to wrong clusters[1]. In addition to the silhouette plot, new versions with different Ks and random initialization seeds of the clustering in the t-SNE map are shown (Supplementary Fig. 15). As seen, similar results as was presented in this work can easily be reproduced, as the nominated reference protein candidates were for all tested Ks and random initializations clustered together (compare with Supplementary Fig. 16).

Statistical models

Linear regression

A linear regression model assumes a linear relationship between a dependent variable y and independent variables x_n:

$$y_i = \beta_0 + \sum_{n=1}^{N} \beta_n x_{in} + \varepsilon_i. \quad (1)$$

for the ith observation where i varies from 1 to the number of samples available (in our case usually number of participants), where N is the number of independent variables, β_0 is the intercept and ε is the error term. In this work, β-coefficients were used as a metric for strength (absolute value) and direction (sign) of an association between standardized variables.

Logistic regression

A logistic regression model assumes a linear relationship between the log-odds of an event and independent variables x_n:

$$p(x_i) = \frac{1}{1+\exp(-(\beta_0 + \sum_{n=1}^{N} \beta_n x_{in}))}. \quad (2)$$

for the ith observation where i varies from 1 to the number of samples available (in our case usually number of participants), where p is the probability, N is the number of independent variables, β_0 is the log odds when all independent variables are 0. β-coefficients explain change in log-odds for a 1-unit difference in corresponding x_n.

Supplementary Results

Changes in results when adjusting for reference proteins

In this section, examples of CSF relationships that are strengthened or weakened when adjusting for reference proteins are given. This was done by exploring the available CSF protein data in BF1 and BF2, with analyses often partially or fully inspired from findings in previous publications. The analysis was aimed to contribute to further understanding of CSF biomarkers and how reference proteins can affect certain results. In all examples, either Aβ40, NTRK3 or CBLN4 was used as a reference protein.

NeuroToolKit correlations

For this analysis, the relationships of ten established biomarkers measured on an Elecsys® platform (NeuroToolKit assay panel) in BF2 and BF1 were evaluated. This was done by examining the partial correlation (Pearson) adjusting for age, sex, and a potential reference protein. All biomarkers that were evaluated are summarized in Supplementary Tab. 10, where their association with the mean CSF level is given (sorted in decreasing order). Correlation matrices can be seen in Supplementary Fig. 12, where the effect of adjusting for Aβ40 or NTRK3 in the full datasets and for cognitively unimpaired Aβ-negative participants are provided. The correlation matrices show how several correlations were decreased or increased when adjusting for a reference protein. Decreased correlations were most clearly seen for proteins highly associated with the mean CSF level (top rows).

*APOE*4 genotype vs protein expression

The number of apolipoprotein E (*APOE*) ε4 alleles is highly associated with the protein expression of ApoE4. Measurements of ApoE4 CSF protein concentrations were performed using high resolution parallel reaction monitoring mass spectrometry in BF1 for participants with one or two ε4 alleles. Details about the methodology can be found in [2]. Adjusting for a reference protein strengthens the association between the number of ε4 alleles and protein expression of ApoE4, as can be seen in Supplementary Tab. 6.

CSF pQTL Analysis (association of genetic variants and proteins)

In protein quantitative trait loci (pQTL) analyses, associations between certain CSF proteins and genetic variants (Single Nucleotide Polymorphism, SNP) have been identified. Such an analysis has previously been performed on the BF1 cohort, see [3]. There, it was shown that brain volumetric measures may be a confounder for CSF trans-pQTL associations of the GMNC-OSTN region, hypothesized to be due to dilution effects on proteins. To further investigate the effect of adjusting for a reference protein for such pQTL associations, five CSF protein associations with genes of the GMNC-OSTN region from the mentioned article were selected. The associations were highly significant when not adjusting for a reference. All proteins that were evaluated are summarized in Supplementary Tab. 7, where their association with the mean CSF level is provided.

The proteins were evaluated in linear regression models, with the CSF protein as outcome and SNP as main predictor. In all models, age, sex, dementia diagnosis and ten genetic principal components were adjusted for. The change in association when also adjusting for a reference protein (Aβ40 or NTRK3) was the measure of interest. The changes in β-coefficients, P-values and R-squared were used to compare the effect of genotype between different models. The result can be seen in Supplementary Tab. 8. In general, the trans-pQTL associations of the GMNC-OSTN were severely weakened/disappeared when adjusting for a reference protein. This trend was stronger when adjusting for NTRK3 than Aβ40, emphasizing NTRK3s potential as a potential reference protein that can generalize over many CSF biomarker applications.

An additional analysis was performed for the most common CSF *trans*-pQTL variant **rs71635338** testing its association with all available CSF OLINK proteins in BF1 (n=368 when excluding the reference protein NTRK3), see Supplementary Fig 13. The results confirmed that adjusting for NTRK3 as a reference protein influenced not only specific proteins, but almost all tested proteins' relationship to this SNP diminished.

Adjusting for a Reference Protein in P-tau181 Applications

Several articles have been evaluating associations between P-tau181 and other CSF proteins. In addition, CSF P-tau181 has been used in AT(N) grouping to compare CSF protein levels between groups. As has been shown in this work, the properties of P-tau181 are affected when adjusting for a reference protein. Here, we aim to exemplify a few cases where adjusting for a reference protein affects the result of previous work. The proteins that were evaluated were included in Supplementary Tab. 10 where their association with the mean CSF level is described.

sTREM2, sAXL, sTyro3 and YKL-40 association with P-tau181 in NC, SCD and MCI

In several articles, AT(N) grouping has been performed to compare inter-group differences for CSF proteins. Examples are sTREM2[4], sAXL[5] and sTyro3[5]. A-grouping has been done using CSF Aβ42/Aβ40, and T-grouping using CSF P-tau181 without adjusting for any reference proteins. It is likely that there will be a bias of elevated levels of proteins in T+ groups and decreased levels of proteins in T- groups if no reference protein is adjusted for. The reference protein effect is visualized by grouping sTREM2 (Supplementary Fig. 9), sAXL (Supplementary Fig. 10) and sTyro3 (Supplementary Fig. 11) into AT(N) groups and compare group differences for the BF2 cohort of participants with NC, SCD or MCI. Here, T-grouping has been performed in five ways: CSF without reference (P-tau cutoff 21.8 pg/ml as in [4]), CSF with reference protein Aβ40 (logistic regression, cutoff: $CSF\ P\text{-}tau181 > 8.14 + 1.20c_{A\beta40}$), CSF with reference protein NTRK3 (logistic regression, cutoff: $CSF\ P\text{-}tau181 > -6.10 + 11.6c_{NTRK3}$), CSF with a reference protein CBLN4 (logistic regression, cutoff: $CSF\ P\text{-}tau181 > 40.3 + 9.27c_{CBLN4}$) and PET. If using CSF grouping, adjusting for a reference protein reduced the group differences, creating a better concordance between CSF and PET grouping and removing strong correlations that seem to have appeared due to mean CSF protein levels.

In addition, the three proteins' association with P-tau181 were evaluated in linear regression models (adjusted for age and sex). Here, the protein YKL-40 was also evaluated as it has been found to be highly associated with P-tau181 in [6], both for Aβ- and Aβ+ participants. As seen in Supplementary Tab. 4, the effects of the associations were severely reduced or even disappeared when adjusting for a reference protein. This

was particularly evident for sTREM2, sAXL and sTyro3, whereas for YKL-40 the relationship was reduced in significance but still relatively strong.

α-synuclein in AD Dementia

In previous research, CSF α-synuclein has been found be highly associated with CSF P-tau181 in AD dementia participants, see for example Majbour et al. and Slaets et al.[7,8] Therefore, a similar analysis as in Supplementary Tab. 4 was performed for CSF α-synuclein in BF2, but here only for participants with AD dementia, see Supplementary Tab. 5. Again, the effect size of the association decreases when adjusting for reference proteins.

Supplementary Note 1: Reference Protein Candidate Profiles

This section describes the seven reference protein candidates (NTRK3, NTRK2, BLMH, CBLN4, PTPRN2, PTPRS and Aβ40) from a biological perspective. This analysis is fully based on protein descriptions in the Human Protein Atlas[9–11] (website: www.proteinatlas.org).

Neurotrophic receptor tyrosine kinase 3 (NTRK3)

NTRK3 is a membrane-bound receptor that phosphorylates itself and members of the MAPK pathway after neurotrophin binding. Its signaling controls cell survival and differentiation, mainly in neurons. NTRK3 has enhanced specificity in brain tissue but low regional brain specificity. It is an intracellular and membrane bound protein, expressed in the nucleus and cytoplasm. Data characteristics in BF2 can be seen in Supplementary Fig. 22, 23 and Supplementary Tab. 11.

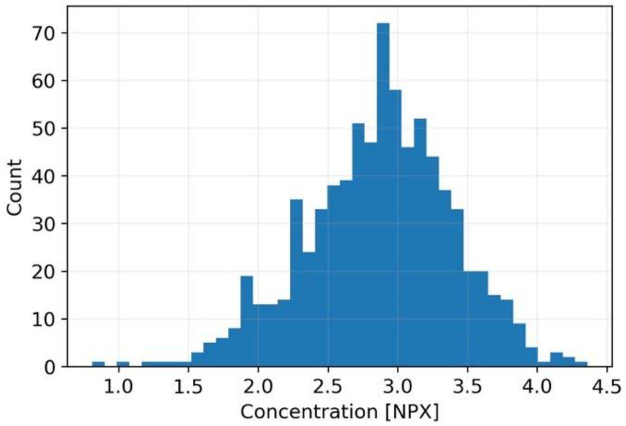

Supplementary Figure 22: NTRK3 concentration distribution. Distribution of protein concentration in BF2 training data.

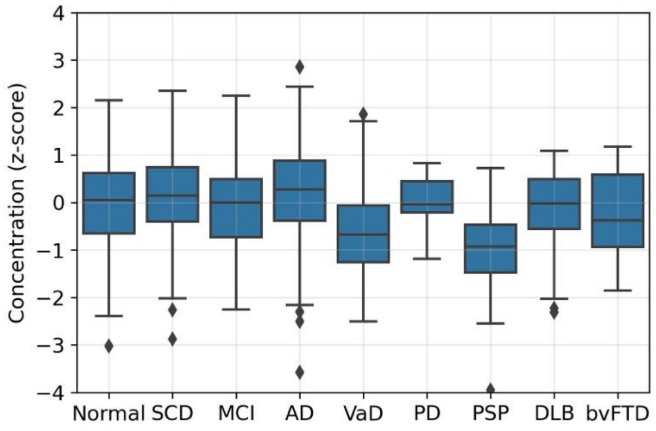

Supplementary Figure 23: NTRK3 diagnostic group boxplot. Distribution of standardized protein concentration between diagnostic groups (>10 individuals) for BF2 training data. The box shows the quartiles of the dataset with the median as the line within. The whiskers extend to show the rest of the distribution with single points as outliers. Number of participants in each group: normal cognition: n = 196, subjective cognitive decline (SCD): n = 98, mild cognitive impairment (MCI): n = 50, Alzheimer's disease (AD): n = 237, vascular dementia (VaD): n = 26, Parkinson's disease (PD): n = 12, Progressive supranuclear palsy (PSP): n = 17, dementia with Lewy bodies (DLB): n = 30, behavioral variant of Frontotemporal dementia (bvFTD): n = 21.

Supplementary Table 11: NTRK3 protein data info. Analysis info from OLINK measures. Association (from linear regression model) with the main predictor to estimate suitability as reference protein for this biomarker. AUC without main predictor to estimate reference candidate's predictive power without main biomarker. All adjusted for age and sex. An optimal reference should be associated with the main predictor while not being highly predictive of the outcome without the main predictor.

**Association with mean CSF level**	
**Linear regression**	$\beta = 0.73$, p<1e-130
**Partial correlation (Pearson)**	0.78
**OLINK analysis info**	
Panel	Neurology
Mean LOD [NPX]	-5.0
Missing Frequency [%]	0
**CSF P-tau181 → Tau PET**	
Association with main predictor	$\beta = 0.48$, p<1e-40
Mean AUC without main predictor	0.62
**CSF Aβ42 → Aβ PET**	
Association with main predictor	$\beta = 0.44$, p<1e-30
Mean AUC without main predictor	0.65

Neurotrophic receptor tyrosine kinase 2 (NTRK2)

NTRK2 is a membrane-bound receptor that phosphorylates itself and members of the MAPK pathway after neurotrophin binding. Its signaling controls cell survival, proliferation, migration, synapse formation and differentiation, mainly in neurons. NTRK3 has enhanced specificity in brain (and thyroid gland) tissue but low regional brain specificity. It is a membrane bound protein, expressed in the cytoplasm. Data characteristics in BF2 can be seen in Supplementary Fig. 24, 25 and Supplementary Tab. 12.

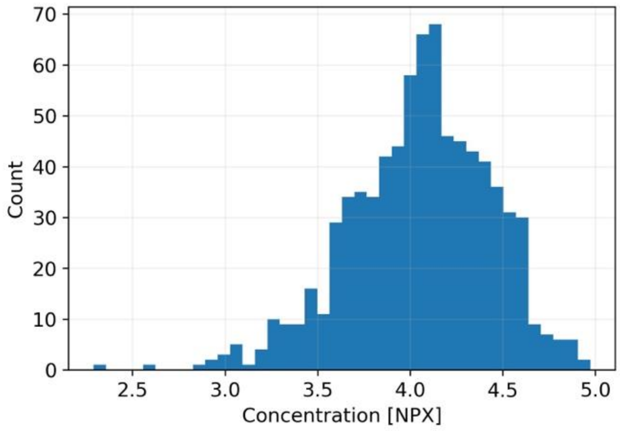

Supplementary Figure 24: NTRK2 concentration distribution. Distribution of protein concentration in BF2 training data.

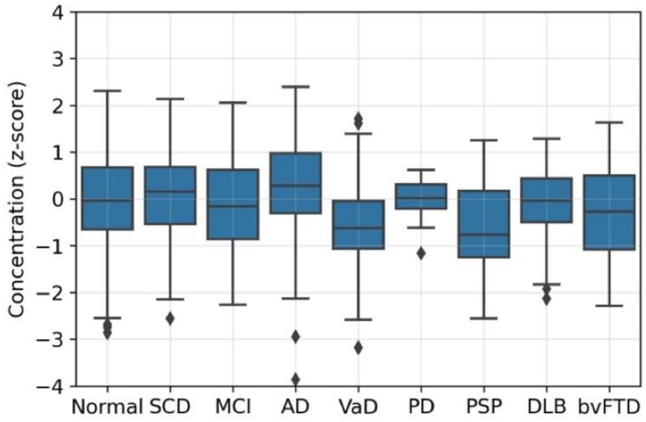

Supplementary Figure 25: NTRK2 diagnostic group boxplot. Distribution of standardized protein concentration between diagnostic groups (>10 individuals) for BF2 training data. The box shows the quartiles of the dataset with the median as the line within. The whiskers extend to show the rest of the distribution with single points as outliers. Number of participants in each group: normal cognition: n = 196, subjective cognitive decline (SCD): n = 98, mild cognitive impairment (MCI): n = 50, Alzheimer's disease (AD): n = 237, vascular dementia (VaD): n = 26, Parkinson's disease (PD): n = 12, Progressive supranuclear palsy (PSP): n = 17, dementia with Lewy bodies (DLB): n = 30, behavioral variant of Frontotemporal dementia (bvFTD): n = 21.

Supplementary Table 12: NTRK2 protein data info. Analysis info from OLINK measures. Association (from linear regression model) with the main predictor to estimate suitability as reference protein for this biomarker. AUC without main predictor to estimate reference candidate's predictive power without main biomarker. All adjusted for age and sex. An optimal reference should be associated with the main predictor while not being highly predictive of the outcome without the main predictor.

**Association with mean CSF level**	
**Linear regression**	β = 0.66, p<1e-100
**Partial correlation (Pearson)**	0.71
**OLINK analysis info**	
Panel	Cardiometabolic
Mean LOD [NPX]	-2.6
Missing Frequency [%]	0
**CSF P-tau181 → Tau PET**	
Association with main predictor	β = 0.50, p<1e-40
Mean AUC without main predictor	0.62
**CSF Aβ42 → Aβ PET**	
Association with main predictor	β = 0.40, p<1e-20
Mean AUC without main predictor	0.66

Bleomycin hydrolase (BLMH)

BLMH is a cytoplasmic cysteine peptidase that is highly conserved through evolution. Its normal physiological role is unknown. BLMH has enhanced specificity in skin tissue and has low regional brain specificity. It is an intracellular protein expressed in the cytoplasm. Data characteristics in BF2 can be seen in Supplementary Fig. 26, 27 and Supplementary Tab. 13.

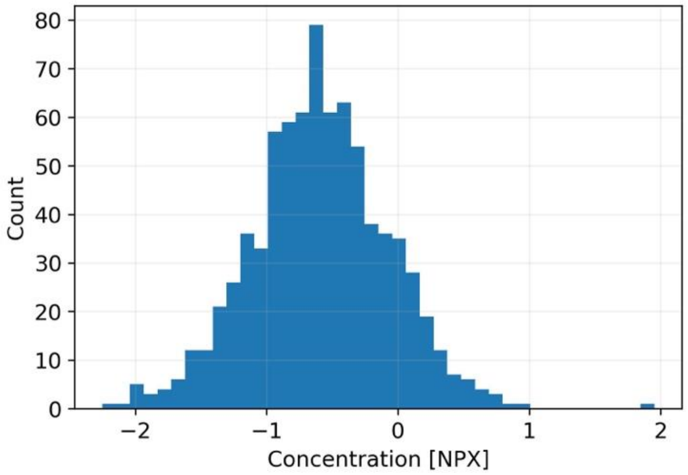

Supplementary Figure 26: BLMH concentration distribution. Distribution of protein concentration in BF2 training data.

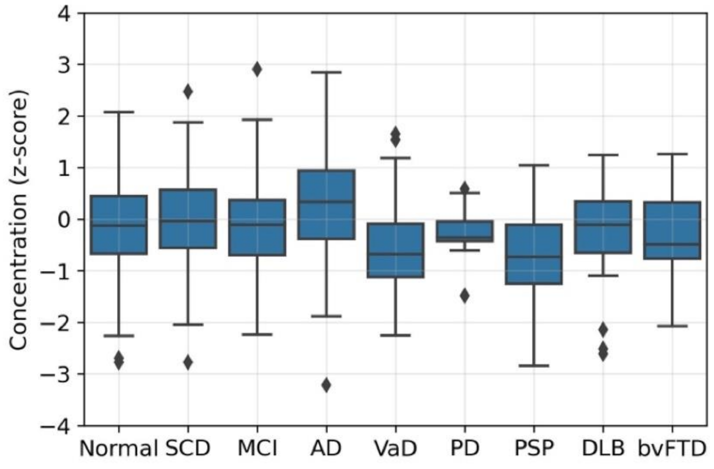

Supplementary Figure 27: BLMH diagnostic group boxplot. Distribution of standardized protein concentration between diagnostic groups (>10 individuals) for BF2 training data. The box shows the quartiles of the dataset with the median as the line within. The whiskers extend to show the rest of the distribution with single points as outliers. Number of participants in each group: normal cognition: n = 196, subjective cognitive decline (SCD): n = 98, mild cognitive impairment (MCI): n = 50, Alzheimer's disease (AD): n = 237, vascular dementia (VaD): n = 26, Parkinson's disease (PD): n = 12, Progressive supranuclear palsy (PSP): n = 17, dementia with Lewy bodies (DLB): n = 30, behavioral variant of Frontotemporal dementia (bvFTD): n = 21.

Supplementary Table 13: BLMH protein data info. Analysis info from OLINK measures. Association (from linear regression model) with the main predictor to estimate suitability as reference protein for this biomarker. AUC without main predictor to estimate reference candidate's predictive power without main biomarker. All adjusted for age and sex. An optimal reference should be associated with the main predictor while not being highly predictive of the outcome without the main predictor.

**Association with mean CSF level**	
**Linear regression**	β = 0.67, p<1e-100
**Partial correlation (Pearson)**	0.72
**OLINK analysis info**	
Panel	Cardiometabolic
Mean LOD [NPX]	-6.2
Missing Frequency [%]	0
**CSF P-tau181 → Tau PET**	
Association with main predictor	β = 0.56, p<1e-60
Mean AUC without main predictor	0.64
**CSF Aβ42 → Aβ PET**	
Association with main predictor	β = 0.33, p<1e-15
Mean AUC without main predictor	0.67

Cerebellin 4 precursor (CBLN4)

CBLN4 is a synaptic organizer that is involved in regulation of neurexin signaling during synapse development. The protein has enhanced specificity in adrenal gland, brain and epididymis tissue. It has low regional brain specificity. Cellular location mainly in synapses in neurons, and extracellularly secreted in brain. Data characteristics in BF2 can be seen in Supplementary Fig. 28, 29 and Supplementary Tab. 14.

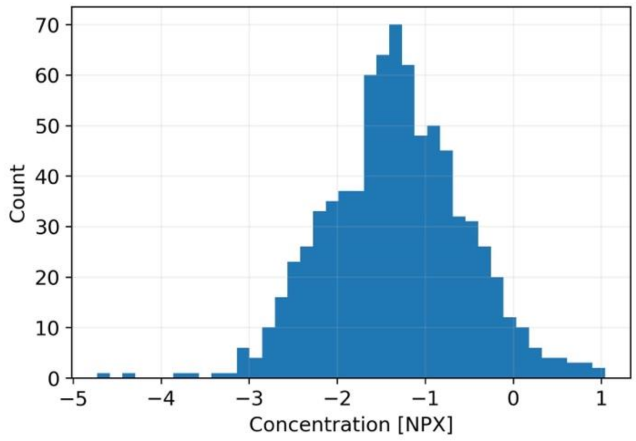

Supplementary Figure 28: CBLN4 concentration distribution. Distribution of protein concentration in BF2 training data.

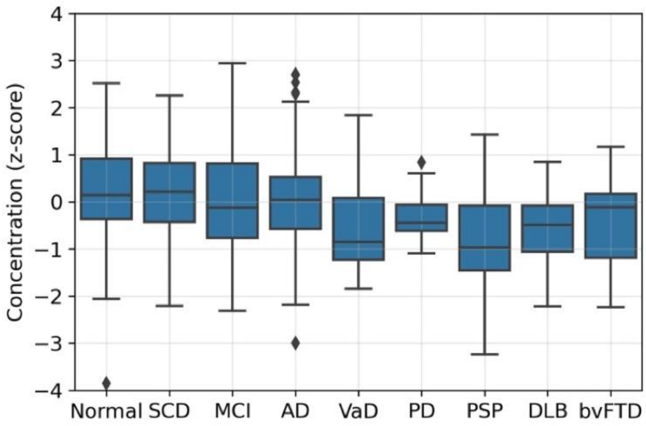

Supplementary Figure 29: CBLN4 diagnostic group boxplot. Distribution of standardized protein concentration between diagnostic groups (>10 individuals) for BF2 training data. The box shows the quartiles of the dataset with the median as the line within. The whiskers extend to show the rest of the distribution with single points as outliers. Number of participants in each group: normal cognition: n = 196, subjective cognitive decline (SCD): n = 98, mild cognitive impairment (MCI): n = 50, Alzheimer's disease (AD): n = 237, vascular dementia (VaD): n = 26, Parkinson's disease (PD): n = 12, Progressive supranuclear palsy (PSP): n = 17, dementia with Lewy bodies (DLB): n = 30, behavioral variant of Frontotemporal dementia (bvFTD): n = 21.

Supplementary Table 14: CBLN4 protein data info. Analysis info from OLINK measures. Association (from linear regression model) with the main predictor to estimate suitability as reference protein for this biomarker. AUC without main predictor to estimate reference candidate's predictive power without main biomarker. All adjusted for age and sex. An optimal reference should be associated with the main predictor while not being highly predictive of the outcome without the main predictor.

**Association with mean CSF level**	
**Linear regression**	β = 0.56, p<1e-70
**Partial correlation (Pearson)**	0.62
**OLINK analysis info**	
Panel	Oncology
Mean LOD [NPX]	-4.5
Missing Frequency [%]	0.0008
**CSF P-tau181 → Tau PET**	
Association with main predictor	β = 0.44, p<1e-30
Mean AUC without main predictor	0.64
**CSF Aβ42 → Aβ PET**	
Association with main predictor	β = 0.47, p<1e-40
Mean AUC without main predictor	0.65

Protein tyrosine phosphatase receptor type N2 (PTPRN2)

PTPRN2 is involved in the vesicle-mediated secretory processes, where it is required for normal accumulation of secretory vesicles in hippocampus, pituitary and pancreatic islets. The protein has enhanced specificity in brain and pancreas tissue. It has low regional brain specificity and is expressed in the brain cells' cytoplasm. PTPRN2 is an intracellular and membrane bound protein. Data characteristics in BF2 can be seen in Supplementary Fig. 30, 31 and Supplementary Tab. 15.

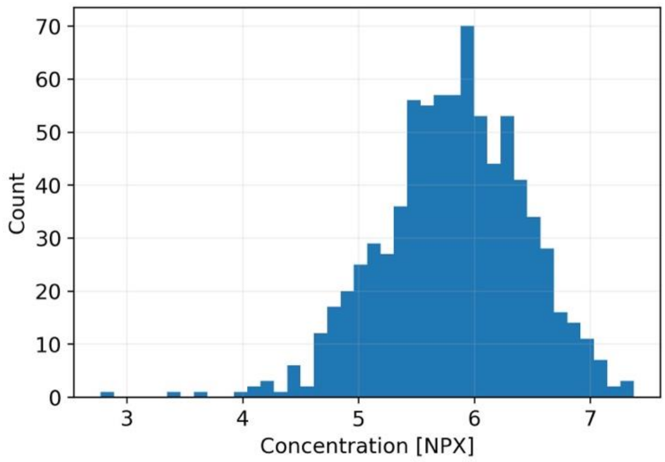

Supplementary Figure 30: PTPRN2 concentration distribution. Distribution of protein concentration in BF2 training data.

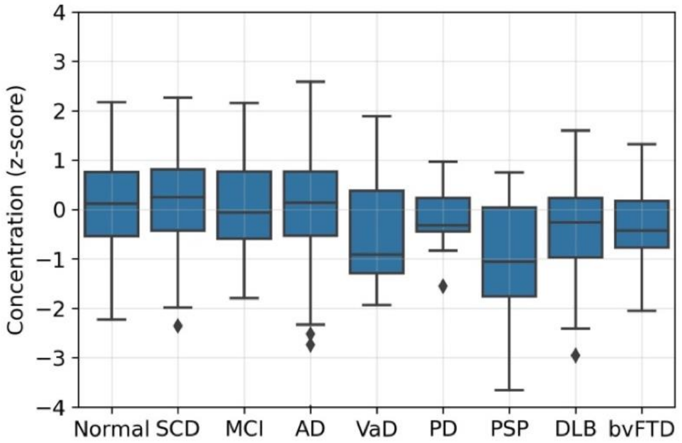

Supplementary Figure 31: PTPRN2 diagnostic group boxplot. Distribution of standardized protein concentration between diagnostic groups (>10 individuals) for BF2 training data. The box shows the quartiles of the dataset with the median as the line within. The whiskers extend to show the rest of the distribution with single points as outliers. Number of participants in each group: normal cognition: n = 196, subjective cognitive decline (SCD): n = 98, mild cognitive impairment (MCI): n = 50, Alzheimer's disease (AD): n = 237, vascular dementia (VaD): n = 26, Parkinson's disease (PD): n = 12, Progressive supranuclear palsy (PSP): n = 17, dementia with Lewy bodies (DLB): n = 30, behavioral variant of Frontotemporal dementia (bvFTD): n = 21.

Supplementary Table 15: PTPRN2 protein data info. Analysis info from OLINK measures. Association (from linear regression model) with the main predictor to estimate suitability as reference protein for this biomarker. AUC without main predictor to estimate reference candidate's predictive power without main biomarker. All adjusted for age and sex. An optimal reference should be associated with the main predictor while not being highly predictive of the outcome without the main predictor.

**Association with mean CSF level**	
**Linear regression**	β = 0.65, p<1e-100
**Partial correlation (Pearson)**	0.71
**OLINK analysis info**	
Panel	Neurology
Mean LOD [NPX]	-3.6
Missing Frequency [%]	0
**CSF P-tau181 → Tau PET**	
Association with main predictor	β = 0.48, p<1e-40
Mean AUC without main predictor	0.63
**CSF Aβ42 → Aβ PET**	
Association with main predictor	β = 0.49, p<1e-40
Mean AUC without main predictor	0.65

Protein tyrosine phosphatase receptor type S (PTPRS)

PTPRS is a cell surface receptor that contributes to the regulation of neurite and axonal outgrowth, differentiation, mitotic cycle and oncogenic transformation. The protein has low tissue specificity and low regional brain specificity. It is mainly expressed in neurons and located in the cytosol and plasma membrane. PTPRS is a membrane bound protein and extracellularly secreted. Data characteristics in BF2 can be seen in Supplementary Fig. 32, 33 and Supplementary Tab. 16.

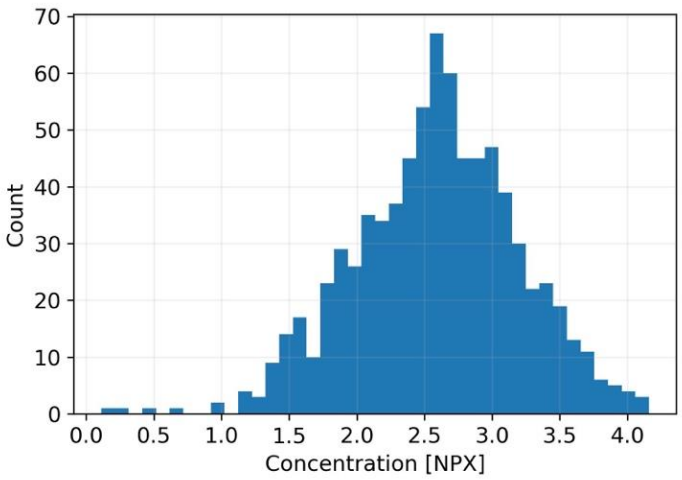

Supplementary Figure 32: PTPRS concentration distribution. Distribution of protein concentration in BF2 training data

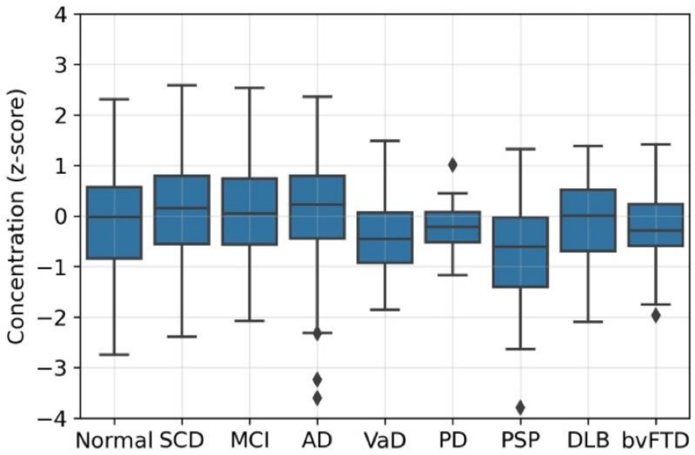

Supplementary Figure 33: PTPRS diagnostic group boxplot. Distribution of standardized protein concentration between diagnostic groups (>10 individuals) for BF2 training data. The box shows the quartiles of the dataset with the median as the line within. The whiskers extend to show the rest of the distribution with single points as outliers. Number of participants in each group: normal cognition: n = 196, subjective cognitive decline (SCD): n = 98, mild cognitive impairment (MCI): n = 50, Alzheimer's disease (AD): n = 237, vascular dementia (VaD): n = 26, Parkinson's disease (PD): n = 12, Progressive supranuclear palsy (PSP): n = 17, dementia with Lewy bodies (DLB): n = 30, behavioral variant of Frontotemporal dementia (bvFTD): n = 21.

Supplementary Table 16: PTPRS protein data info. Analysis info from OLINK measures. Association (from linear regression model) with the main predictor to estimate suitability as reference protein for this biomarker. AUC without main predictor to estimate reference candidate's predictive power without main biomarker. All adjusted for age and sex. An optimal reference should be associated with the main predictor while not being highly predictive of the outcome without the main predictor.

**Association with mean CSF level**	
**Linear regression**	β = 0.76, p<1e-140
**Partial correlation (Pearson)**	0.80
**OLINK analysis info**	
Panel	Neurology
Mean LOD [NPX]	-3.6
Missing Frequency [%]	0
**CSF P-tau181 → Tau PET**	
Association with main predictor	β = 0.48, p<1e-40
Mean AUC without main predictor	0.63
**CSF Aβ42 → Aβ PET**	
Association with main predictor	β = 0.49, p<1e-40
Mean AUC without main predictor	0.65

Amyloid-β40 (Aβ40)

Aβ40 is a peptide derived from the amyloid beta precursor protein (APP). APP is a cell surface receptor that contributes to neurite growth, neuronal adhesion and axonogenesis. It has low tissue specificity and low regional brain specificity. It is a membrane bound protein, mainly expressed in the cytoplasm and secreted extracellularly. Data characteristics in BF2 can be seen in Supplementary Fig. 34, 35 and Supplementary Tab. 17.

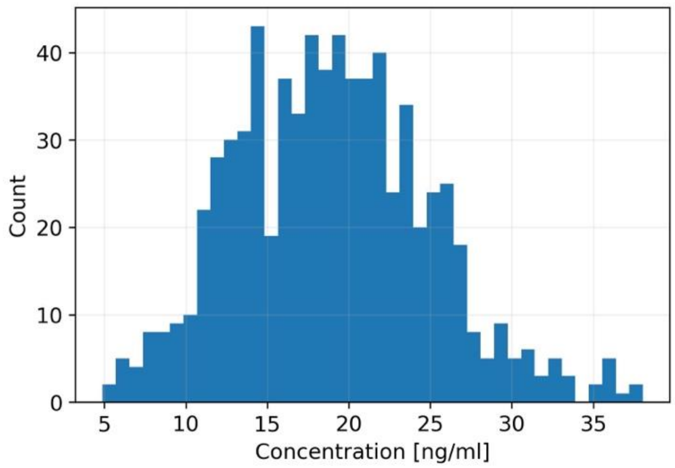

Supplementary Figure 34: Aβ40 concentration distribution. Distribution of protein concentration in BF2 training data.

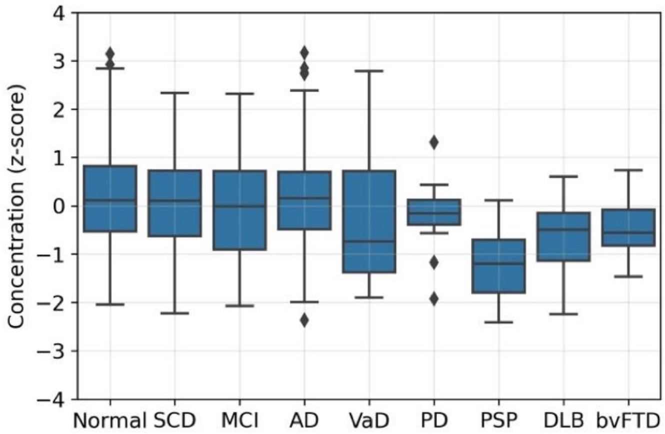

Supplementary Figure 35: Aβ40 diagnostic group boxplot. Distribution of standardized protein concentration between diagnostic groups (>10 individuals) for BF2 training data. The box shows the quartiles of the dataset with the median as the line within. The whiskers extend to show the rest of the distribution with single points as outliers. Number of participants in each group: normal cognition: n = 196, subjective cognitive decline (SCD): n = 98, mild cognitive impairment (MCI): n = 50, Alzheimer's disease (AD): n = 237, vascular dementia (VaD): n = 26, Parkinson's disease (PD): n = 12, Progressive supranuclear palsy (PSP): n = 17, dementia with Lewy bodies (DLB): n = 30, behavioral variant of Frontotemporal dementia (bvFTD): n = 21.

Supplementary Table 17: Aβ40 protein data info. This protein was measured with an ELISA assay, therefore no OLINK info exist. Association (from linear regression model) with the main predictor to estimate suitability as reference protein for this biomarker. AUC without main predictor to estimate reference candidate's predictive power without main biomarker. All adjusted for age and sex. An optimal reference should be associated with the main predictor while not being highly predictive of the outcome without the main predictor.

**Association with mean CSF level**	
**Linear regression**	β = 0.44, p<1e-30
**Partial correlation (Pearson)**	0.48
**CSF P-tau181 → Tau PET**	
Association with main predictor	β = 0.50, p<1e-50
Mean AUC without main predictor	0.62
**CSF Aβ42 → Aβ PET**	
Association with main predictor	β = 0.52, p<1e-40
Mean AUC without main predictor	0.65

Supplementary References

1. Shahapure KR, Nicholas C. Cluster quality analysis using silhouette score. In: *Proceedings - 2020 IEEE 7th International Conference on Data Science and Advanced Analytics, DSAA 2020*. Institute of Electrical and Electronics Engineers Inc.; 2020:747-748. doi:10.1109/DSAA49011.2020.00096

2. Minta K, Brinkmalm G, Janelidze S, et al. Quantification of total apolipoprotein e and its isoforms in cerebrospinal fluid from patients with neurodegenerative diseases. *Alzheimers Res Ther*. 2020;12(1). doi:10.1186/s13195-020-00585-7

3. Hansson O, Kumar A, Janelidze S, et al. The genetic regulation of protein expression in cerebrospinal fluid. *EMBO Mol Med*. 2023;15(1). doi:10.15252/emmm.202216359

4. Suárez-Calvet M, Morenas-Rodríguez E, Kleinberger G, et al. Early increase of CSF sTREM2 in Alzheimer's disease is associated with tau related-neurodegeneration but not with amyloid-β pathology. *Mol Neurodegener*. 2019;14(1). doi:10.1186/s13024-018-0301-5

5. Brosseron F, Maass A, Kleineidam L, et al. Soluble TAM receptors sAXL and sTyro3 predict structural and functional protection in Alzheimer's disease. *Neuron*. 2022;110(6):1009-1022.e4. doi:10.1016/j.neuron.2021.12.016

6. Janelidze S, Mattsson N, Stomrud E, et al. CSF biomarkers of neuroinflammation and cerebrovascular dysfunction in early Alzheimer disease. *Neurology*. 2018;91(9):e867-e877. doi:10.1212/WNL.0000000000006082

7. Majbour NK, Chiasserini D, Vaikath NN, et al. Increased levels of CSF total but not oligomeric or phosphorylated forms of alpha-synuclein in patients diagnosed with probable Alzheimer's disease. *Sci Rep*. 2017;7. doi:10.1038/srep40263

8. Slaets S, Vanmechelen E, le Bastard N, et al. Increased CSF α-synuclein levels in Alzheimer's disease: Correlation with tau levels. *Alzheimer's and Dementia*. 2014;10(5):S290-S298. doi:10.1016/j.jalz.2013.10.004

9. Uhlén M, Fagerberg L, Hallström BM, et al. Tissue-based map of the human proteome. *Science (1979)*. 2015;347(6220). doi:10.1126/science.1260419

10. Pontén F, Jirström K, Uhlen M. The Human Protein Atlas - A tool for pathology. *Journal of Pathology*. 2008;216(4):387-393. doi:10.1002/path.2440

11. Uhlen M, Oksvold P, Fagerberg L, et al. Towards a knowledge-based Human Protein Atlas. *Nat Biotechnol*. 2010;28(12):1248-1250. doi:10.1038/nbt1210-1248
