# Supplementary material for: Cerebrospinal fluid reference proteins increase accuracy and interpretability of biomarkers for brain diseases
Source: Nat Commun. 2024 May 1;15:3676. doi: 10.1038/s41467-024-47971-5 (PMC11063138; doi:10.1038/s41467-024-47971-5)
Supplement: Supplementary file 3 — Reporting Summary [file 41467_2024_47971_MOESM3_ESM.pdf]

nature portfolio

Corresponding author(s): Linda Karlsson; Oskar Hansson

Last updated by author(s): Apr 5, 2024

Reporting Summary

Nature Portfolio wishes to improve the reproducibility of the work that we publish. This form provides structure for consistency and transparency in reporting. For further information on Nature Portfolio policies, see our Editorial Policies and the Editorial Policy Checklist.

Statistics

For all statistical analyses, confirm that the following items are present in the figure legend, table legend, main text, or Methods section.

n/a	Confirmed	
☐	☒	The exact sample size (*n*) for each experimental group/condition, given as a discrete number and unit of measurement
☐	☒	A statement on whether measurements were taken from distinct samples or whether the same sample was measured repeatedly
☐	☒	The statistical test(s) used AND whether they are one- or two-sided<br>*Only common tests should be described solely by name; describe more complex techniques in the Methods section.*
☐	☒	A description of all covariates tested
☐	☒	A description of any assumptions or corrections, such as tests of normality and adjustment for multiple comparisons
☐	☒	A full description of the statistical parameters including central tendency (e.g. means) or other basic estimates (e.g. regression coefficient) AND variation (e.g. standard deviation) or associated estimates of uncertainty (e.g. confidence intervals)
☐	☒	For null hypothesis testing, the test statistic (e.g. *F*, *t*, *r*) with confidence intervals, effect sizes, degrees of freedom and *P* value noted<br>*Give P values as exact values whenever suitable.*
☒	☐	For Bayesian analysis, information on the choice of priors and Markov chain Monte Carlo settings
☒	☐	For hierarchical and complex designs, identification of the appropriate level for tests and full reporting of outcomes
☐	☒	Estimates of effect sizes (e.g. Cohen's *d*, Pearson's *r*), indicating how they were calculated

Our web collection on statistics for biologists contains articles on many of the points above.

Software and code

Policy information about availability of computer code

Data collection	No software was used.
Data analysis	Code can be found in the GIT repository https://github.com/karlssonlinda/reference_protein_project.The repository has been linked to Zenodo with release v1.0.0. Python version 3.9 (packages: sklearn 0.0, pandas 1.4.4, numpy 1.23.3, matplotlib 3.5.3, pingouin 0.5.3, tqdm 4.64.1,statsmodels 0.13.2) and R version 4.2 (packages: tidyverse 1.3.2, pROC 1.18.0, EWCE 1.4.0). Seurat version 4.3.0 and WEB-based Gene SeT AnaLysis Toolkit (WebGestalt) 2019 were also used.

For manuscripts utilizing custom algorithms or software that are central to the research but not yet described in published literature, software must be made available to editors and reviewers. We strongly encourage code deposition in a community repository (e.g. GitHub). See the Nature Portfolio guidelines for submitting code & software for further information.

Data

Policy information about availability of data

All manuscripts must include a data availability statement. This statement should provide the following information, where applicable:

- Accession codes, unique identifiers, or web links for publicly available datasets
- A description of any restrictions on data availability
- For clinical datasets or third party data, please ensure that the statement adheres to our policy

Pseudonymized BioFINDER-1 and BioFINDER-2 data can be shared to qualified academic researchers after request (PI:OH) for the purpose of replicating procedures and results presented in the study. Data transfer must be performed in agreement with EU legislation regarding general data protection regulation and decisions by

the Ethical Review Board of Sweden and Region Skåne. Human MTG 10x SEA-AD Allen Brain data from 202260 are publicly available and can be downloaded from celltypes.brain-map.org/rnaseq. Tissue datasets from the Human Protein Atlas are also publicly available and can be downloaded from https://www.proteinatlas.org/about/download.

Research involving human participants, their data, or biological material

Policy information about studies with human participants or human data. See also policy information about sex, gender (identity/presentation), and sexual orientation and race, ethnicity and racism.

Reporting on sex and gender	We used the term "sex" throughout the manuscript. Sex was determined based on self-reporting. Statistical analyses included sex as a covariate; the study included 2 independent cohorts altogether comprised of balanced numbers of males (n=389 in BioFINDER-1 and 438 in BioFINDER-2) and females (n=515 in BioFINDER-1 and 392 in BioFINDER-2); therefore, we believe the findings apply to both sexes.
Reporting on race, ethnicity, or other socially relevant groupings	This study did not include categorization of race, ethnicity and/or other socially relevant groupings.
Population characteristics	Detailed information is given in Table 1. In short, we present results for analyses from the BioFINDER-1 (mean age 73 years [SD 5.5]) and BioFINDER-2 (mean age 69 years [SD 12]) cohorts, both with similar demographics. Participants were included based on baseline (cross-sectional) CSF measures (OLINK + Aβ40) in the present study. Both cohorts consisted of individuals with either normal cognition (NC), subjective cognitive decline (SCD), mild cognitive impairment (MCI), dementia or another neurodegenerative disease. For participants with longitudinal data, these were used to assess conversion to AD dementia (based on the treating physician's assessments).
Recruitment	This project was done as part of the prospective Swedish BioFINDER study. All patients were recruited from the Southern part of Sweden and underwent baseline examination from 2007 to 2015 (BioFINDER-1) or from 2017 to 2021 (BioFINDER-2). In BioFINDER-1, participants were consecutively recruited based on referrals (mostly from primary care) to participating memory clinics (in the towns of Malmö, Lund and Ängelholm in Sweden). In BioFINDER-2, patients were included after being referred to the memory clinic of Skåne University Hospital in Malmö, Sweden.
Ethics oversight	The study was approved by the Swedish Ethical Review Authority. All participants gave their informed consent to participate in the study and the data were collected according to the Declaration of Helsinki.

Note that full information on the approval of the study protocol must also be provided in the manuscript.

Field-specific reporting

Please select the one below that is the best fit for your research. If you are not sure, read the appropriate sections before making your selection.

[x] Life sciences [ ] Behavioural & social sciences [ ] Ecological, evolutionary & environmental sciences

For a reference copy of the document with all sections, see nature.com/documents/nr-reporting-summary-flat.pdf

Life sciences study design

All studies must disclose on these points even when the disclosure is negative.

Sample size	The study was conducted by maximizing the sample sizes available in regards to the number of participants and CSF proteins in the two cohorts, resulting in n=830 participants in BioFINDER-2 with 2944 measured CSF protein concentrations and n=904 participants in BioFINDER-1 with 369 measured CSF protein concentrations. There is no indication that we were insufficiently powered for these analyses.
Data exclusions	The data were limited to the subsets of the source cohorts with available CSF biomarker data, demographic information (age and sex) and outcome measures (tau-PET, Aβ-PET and conversion to AD dementia). See flowchart in Fig. 1.
Replication	To assure generalizability of the suggested reference protein candidates, all exploratory work was performed on a training dataset (80% of BioFINDER-2, n=658) using 10-fold cross-validation, and then evaluated on a held out test set (20%, n=172), where significance testing was done with bootstrap-resampling with replacement (number of iterations = 2000). All suggested reference protein candidates outperformed using no reference protein and using the mean standardized CSF protein level as reference in three models. Furthermore, four reference proteins were validated in the independent BioFINDER-1 cohort, where three of the four suggested reference proteins (Aβ40, NTRK3 and NTRK2) showed to be superior to using no reference protein and using the mean standardized CSF protein level as reference in two models. The fourth reference protein (BLMH) showed trend level improvement compared to using no reference protein, as did using the mean standardized CSF protein level. Significance testing was again assessed with bootstrap-resampling with replacement (number of iterations = 2000). All suggested reference protein candidates could not be validated in BioFINDER-1 as they were not measured. As baseline tau-PET data did not exist in BioFINDER-1, the P-tau181→TauPET model was not evaluated on BioFINDER-1.
Randomization	In these two prospective cohort studies (observational studies) no allocation into experimental groups were performed, therefore randomization is not relevant to this study. Statistical analyses were controlled for potential confounding effect of age and sex.
Blinding	CSF analyses and PET analyses were performed by individuals who were blinded to the clinical data. Authors who performed the data preprocessing were blinded to demographic and clinical characteristics of individuals.

Reporting for specific materials, systems and methods

We require information from authors about some types of materials, experimental systems and methods used in many studies. Here, indicate whether each material, system or method listed is relevant to your study. If you are not sure if a list item applies to your research, read the appropriate section before selecting a response.

Materials & experimental systems

n/a	Involved in the study
☐	☒ Antibodies
☒	☐ Eukaryotic cell lines
☒	☐ Palaeontology and archaeology
☒	☐ Animals and other organisms
☐	☒ Clinical data
☒	☐ Dual use research of concern
☒	☐ Plants

Methods

n/a	Involved in the study
☒	☐ ChIP-seq
☒	☐ Flow cytometry
☒	☐ MRI-based neuroimaging

Antibodies

Antibodies used

CSF samples were analyzed with validated, highly sensitive and specific Proximity Extension Assay (PEA) developed by OLINK Proteomics (Uppsala, Sweden). For BioFINDER-2, the full OLINK Explore 3072 library was used, resulting in eight Proseek Multiplex panels (Oncology I and II, Neurology I and II, Cardiometabolic I and II, Inflammation I and II) to measure the concentration of 2,943 CSF proteins. Each panel contained 367-369 proteins. For BioFINDER-1, four panels (Neurology-exploratory, Neurology-I, Inflammation-I and Cardiovascular-III) were used to measure the concentration of 368 CSF proteins. Each panel contained 92 proteins. All 368 proteins from the BioFINDER-1 panels were also included in the BioFINDER-2 Explore 3072 panels.

CSF biomarkers from the NeuroToolKit assay panel (P-tau181, Aβ42, Aβ40, sTREM2, YKL-40, GFAP, neurogranin, T-tau, S100, alpha synuclein and NfL) were measured in both cohorts using Elecsys assays in accordance with the manufacturer's instructions (Roche Diagnostics International Ltd).

Validation

Proximity Extension Assay (PEA) developed by OLINK Proteomics is a validated, highly sensitive and specific methodology. It has previously been used and described in for example del Campo et al. (Nat Aging. 2022;2:1040–1053) and Vromen et al. (Alzheimer's & Dementia. 2022;8(1):e12240. The NeuroToolKit assay panel is a well-established and highly validated method to generate high quality, reproducible, and comparable biomarker data. It has previously been used and described in for example Milà-Alomà et al. (Alzheimer's & Dementia. 2020;16(10):1358-1371) and Van Hulle et al. (Alzheimer's & Dementia. 2021;17(3):431–445).

Clinical data

Policy information about clinical studies

All manuscripts should comply with the ICMJEguidelines for publication of clinical research and a completedCONSORT checklist must be included with all submissions.

Clinical trial registration

NCT01208675 (BioFINDER-2) and NCT03174938 (BioFINDER-1).

Study protocol

BioFINDER-1: https://clinicaltrials.gov/ct2/show/NCT01208675
BioFINDER-2: https://clinicaltrials.gov/ct2/show/NCT03174938

Data collection

All patients were recruited from the Southern part of Sweden and underwent baseline examination from 2007 to 2015 (BioFINDER-1) or from 2017 to 2021 (BioFINDER-2). Biomarker data was collected at baseline. Data were collected at the Memory Clinic of Skåne university Hospital, Sweden, and the Memory Clinic of Ängelholm Hospital, Sweden.

Outcomes

The main outcome was improvement in sensitivity and specificity of key CSF AD biomarkers when adjusting for a reference protein.
